# Neonatal Clinical Assessment of the Puppy and Kitten: How to Identify Newborns at Risk?

**DOI:** 10.3390/ani14233417

**Published:** 2024-11-26

**Authors:** Keylla Helena Nobre Pacífico Pereira, Kárita da Mata Fuchs, Júlia Cosenza Mendonça, Gleice Mendes Xavier, Diogo Ribeiro Câmara, Raíssa Karolinny Salgueiro Cruz, Maria Lucia Gomes Lourenço

**Affiliations:** 1 Veterinary Neonatology Research Group, Department of Veterinary Clinic, School of Veterinary Medicine and Animal Science, São Paulo State University (UNESP), Botucatu 18618-970, SP, Brazil; keylla_pacifico@hotmail.com (K.H.N.P.P.); karita.fuchs@unesp.br (K.d.M.F.); julia.cosenza@unesp.br (J.C.M.); gleice.xavier@unesp.br (G.M.X.); 2 Department of Veterinary Medicine, Federal University of Alagoas (UFAL), Viçosa 57700-000, AL, Brazil; diogo@vicosa.ufal.br; 3 Department of Veterinary Clinic, Cesmac University Center, Marechal Deodoro 57160-000, AL, Brazil; raissa.cruz@cesmac.edu.br

**Keywords:** neonatology, physical examination, physiology, neonatal triad, canine, feline

## Abstract

Neonatal puppies and kittens have physiological and anatomical characteristics that differ from those of adult animals, which impacts the interpretation of physical examination and complementary tests. The veterinarian must be familiar with the clinical, laboratory, and imaging aspects observed in neonates. It is necessary to thoroughly evaluate the litter and the mother to notice the most subtle signs of illness and advise the owner to constantly monitor the newborns, quickly sending them to specialized care. This review describes how to perform clinical assessments of newborns systematically and recognize clinical signs of neonatal changes or affections in puppies and kittens.

## Simple Summary

Despite advances in puppy and kitten neonatology in recent years, mortality rates for these patients are still high, making the neonatal period a challenge for veterinarians and owners. Early recognition of newborns at risk allows for rapid intervention, increasing the chance of neonatal survival. To this end, an adequate clinical assessment is essential, which demands knowing the particularities of these patients and the reference parameters for age.

## 1. Introduction

Neonatology is a medical area dedicated to newborn care, physiological particularities, and neonatal diseases. Currently, in dogs and cats, this period can be considered between 21 and 30 days of age, when organic systems undergo anatomophysiological maturation that gradually allows neonatal survival without maternal care [1,2]. The characteristics of neonates are completely different from adult animals, which can make the clinical evaluation of these patients challenging.

Newborn care involves multiple aspects (detailed clinical examination of the puppy together with the mother and litter), difficult management, handling, and diagnostic exploration, such as laboratory analyses (sample collection), radiographic, ultrasound, and electrocardiographic examinations, among others, as a consequence of patient size/weight. Therefore, recognizing clinical signs and distinguishing between healthy neonates and sick neonates is often challenging.

Failure in clinical assessment, diagnosis, and puppy and kitten early care is still common, contributing to high mortality rates globally [3,4,5,6,7,8,9]. For a long time, there was a perception that newborns demonstrated nonspecific clinical signs for different diseases with frequent sudden death, leading to thinking that immediate assistance was impossible. Currently, it is known that the clinical signs of a newborn at risk can be noticeable early and that sudden death is preceded by clinical signs (often suggestive/specific of some conditions) that were not identified before, mainly because of the absence of litter monitoring. To perceive clinical signs in newborns, it is necessary to carry out a thorough clinical assessment, knowledge of neonatal particularities, and constant examination of the litter, enabling early diagnosis and treatment that increases the chances of newborn survival.

In addition to the veterinary clinical assessment, the owner also plays an important role in observing clinical signs and identifying puppies at risk. They must be instructed on constant monitoring of the litter, which assessments should be carried out, and when to seek veterinary care.

Despite the importance of neonatology for veterinary medicine, few studies have been published on the physical examination of newborns, and physiological parameters during the neonatal period are incomplete, especially in kittens. The literature on neonatal hematological and biochemical parameters is scarce and mostly outdated or provides limited and insufficient data.

Therefore, knowledge of the neonatal clinical assessment of dogs and cats still has gaps. Understanding the particularities of physiological evolution and changes in clinical parameters during the neonatal period is crucial for the adequate clinical management of these patients. This review addresses how to perform clinical assessments and perceive clinical signs of neonatal disorders in puppies and kittens, as well as providing guidance for the development of future research.

## 2. Methods

The scientific articles used to support this review were selected from the following databases: PubMed/Medline, Scopus, Science Direct, and Google Scholar, based on the search for keywords: Puppy, kitten, physiology, neonatal health, clinical evaluation, vitality scores, neonatal diseases and malformations, neonatal sepsis, neonatal triad, weight, mortality, neonatal hematology and biochemistry, neonatal imaging, neonatal care. The average number of article occurrences for search terms related to the main themes of this manuscript was 400 for neonatal puppies and 300 for neonatal kittens. The selection of articles to be used in the review mainly followed the criteria of describing neonatal parameters during the first weeks of life, related to physiology, development, and the diagnosis of disorders.

## 3. Relevant Sections

### 3.1. Anamnesis

The clinical history of neonatal patients should be obtained by investigating several reproductive aspects related to the parents, pregnancy, parturition, environment, litter, and individual information about each newborn. It is important to highlight that the health of the parents, pregnancy development, and parturition directly influence fetal and neonatal health [2,10]. This means that if we look at the newborn’s diagnostic investigation from a broad point of view, it begins with information acquired “before conception.” The newborn’s health is not defined by circumstances that occur only during the neonatal period, being a continuation of parental genetics, maternal health, pregnancy, and the birth process itself, in addition to all other events that may occur during the neonatal period [11].

Table 1 demonstrates the first stage of the clinical approach and key points to be addressed during neonatal appointments. However, whenever newborns arrive for emergency care, the first step will be to evaluate vital parameters and carry out other procedures to stabilize the patient, followed by a complete medical history and anamnesis.

### 3.2. Newborn Identification

**Figure 1:**
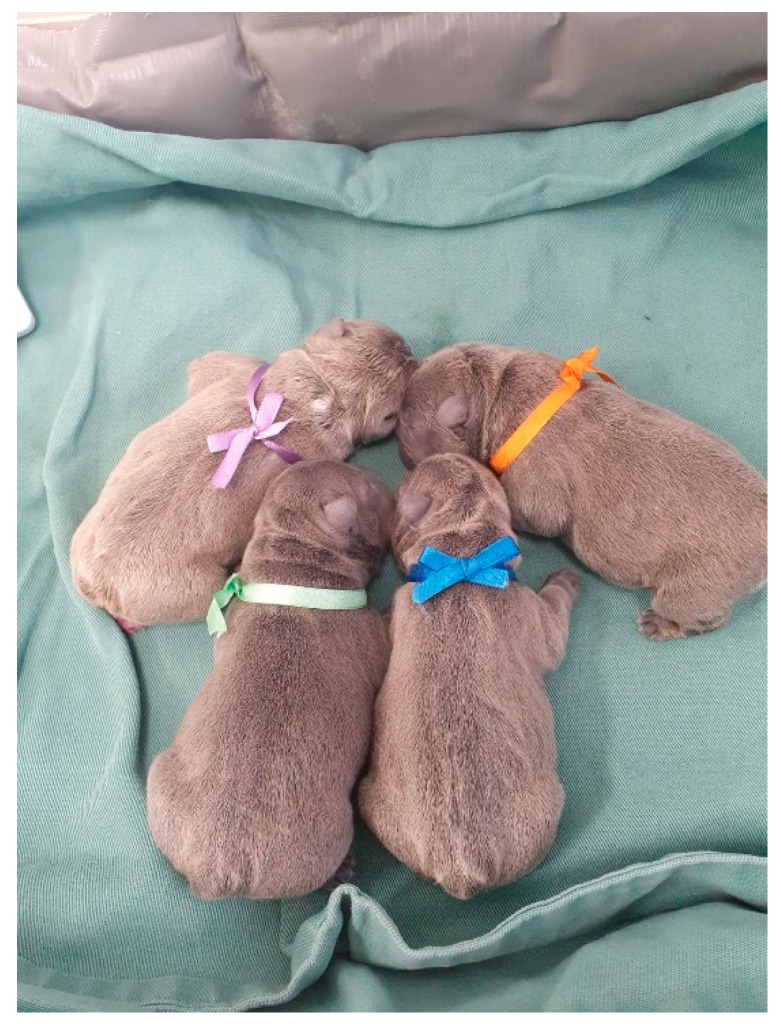
Identification of newborns with colored ribbons around their necks.

The identification of neonates is essential to proceed with clinical appointments since newborns may have similar colored coats and confuse those evaluators. It is necessary to know which newborn the data from the physical examinations and complementary exams belong to or which newborn has already been examined and treated. For identification, colored ribbons (Figure 1), numbered cords around the newborn’s neck, or even bracelets on the limbs can be used.

### 3.3. Physical Examination

**Figure 2:**
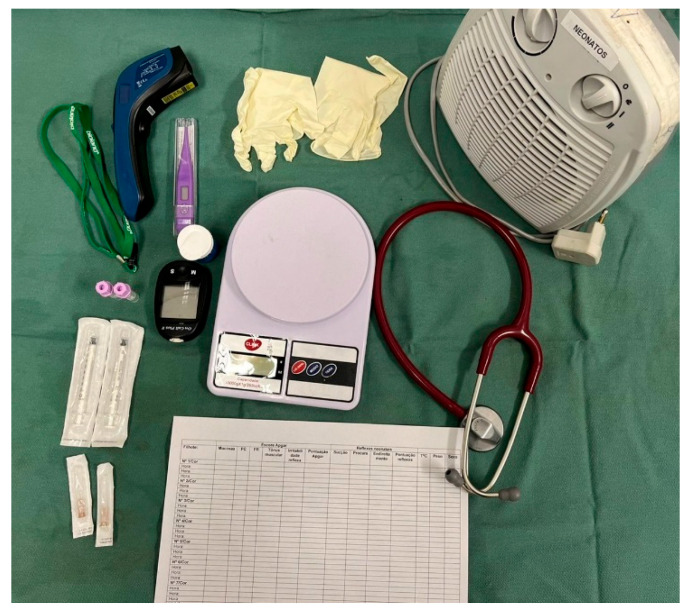
Basic materials and equipment for neonatal clinical assessment.

After identifying and obtaining the clinical history of each newborn in the litter, a systematic physical examination must be conducted. It is essential to know neonatal physiology to correctly interpret the parameters, whose majority differ from those in adults and vary according to the week of age during the neonatal period. The basic materials and equipment needed for neonatal assessment are described in Table 2 and depicted in Figure 2.

During the neonatal period, it is necessary to examine not only the sick newborn but the entire litter and the mother. Although only the affected newborn is often taken for an appointment, the entire litter may be affected. It is also important to observe neonatal changes by comparing the sick sibling with a healthy sibling.

As neonatal affection can be a consequence of maternal disorder, it is essential to carry out a maternal clinical assessment and complementary exams if necessary, due to the possibility of nutritional imbalance, agalactia or hypogalactia, mastitis, abnormal maternal behavior, and changes in uterine status and vaginal secretion [5,6].

**Figure 3:**
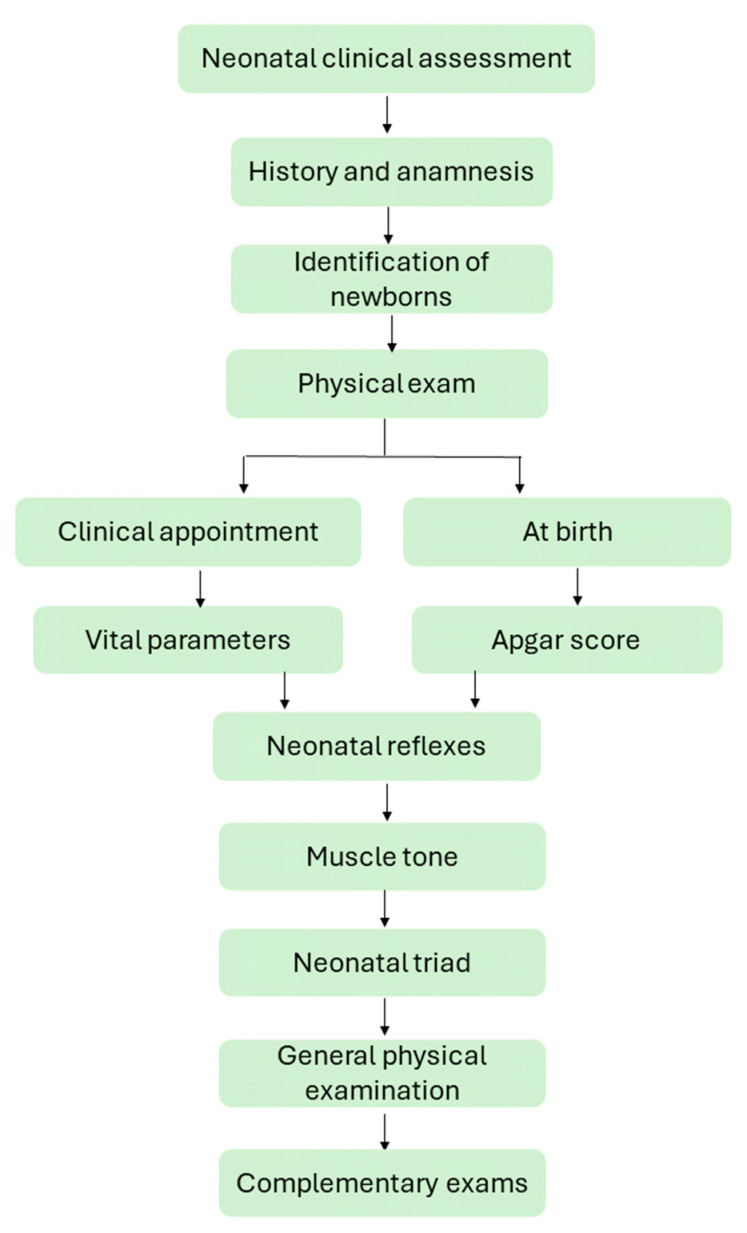
Flowchart of neonatal clinical assessment.

The neonatal physical examination must begin by inspecting the level of consciousness, posture, locomotion, and breathing pattern before manipulation since these parameters can change after touch. Next, the patient’s vital parameters must be assessed, but it must be noted that if the assessment occurs at the birth time, a modified Apgar score should be used. Following, the main neonatal reflexes, muscle tone, neonatal triad, and general physical examination must be performed, evaluating each region of the newborn’s body (Figure 3).

#### 3.3.1. Vital Parameters

Upon clinical appointment, the first step of the physical examination is to evaluate the newborn’s vital parameters, which include heart rate, respiratory rate, body temperature, and blood pressure. However, studies that describe vital parameters in newborns, for the most part, are focused on initial parameters after birth [12,13,14,15,16], with data on the evolution of these parameters over the weeks during the neonatal period being scarce.

Canine and feline neonates have immature cardiovascular systems, with low blood pressure (average of 50–70 mmHg) and low peripheral vascular resistance. To compensate for these aspects and maintain adequate blood perfusion, neonates have a higher heart rate (>200 bpm) and greater cardiac output, plasma volume, and central venous pressure than adults do (5.2 mmHg in neonatal puppies; there are no studies in neonatal kittens) [1,17,18,19,20]. The heart rate gradually decreases, from approximately 260 bpm at birth to 200 bpm in the fourth week of age in puppies (varying with animal size) [14,21] and from 280 bpm at birth to 200 bpm in kittens [16], approaching that of an adult around the seventh to eighth week. On the other hand, there is an increase in systolic blood pressure throughout the neonatal period, from 61 to 70 mmHg at birth to 139 mmHg in the fourth week of life in puppies, and from 50 mmHg at birth to 120 mmHg in the sixth week of life in kittens [1,18,19,20].

**Figure 4:**
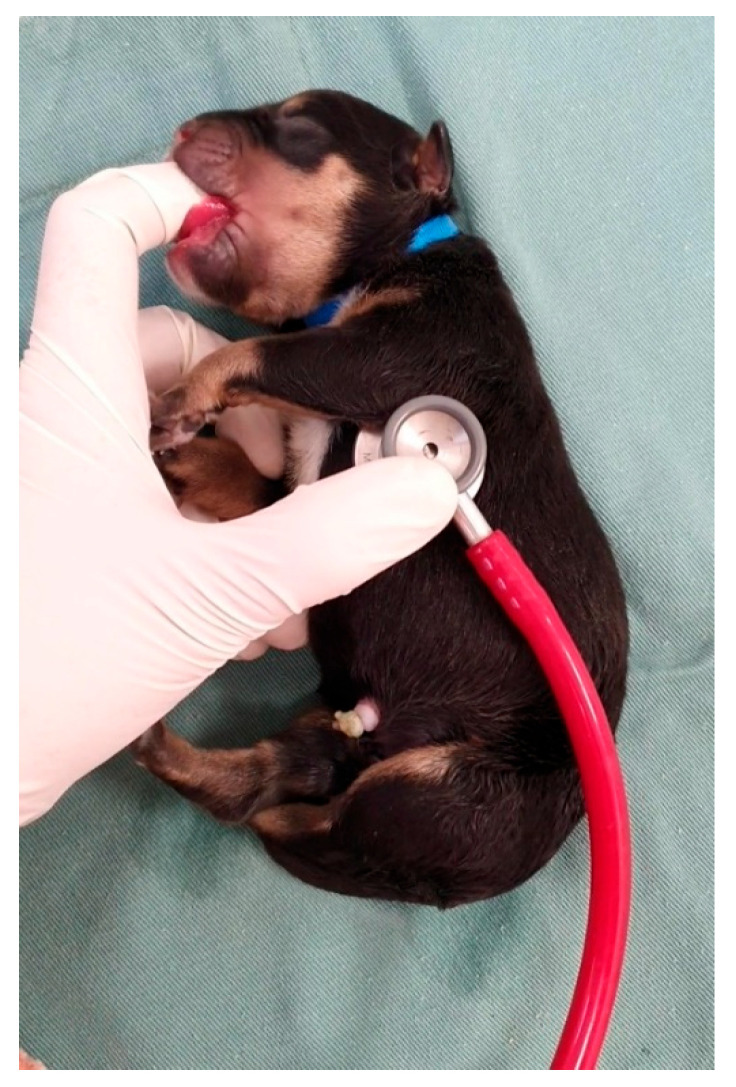
Cardiac auscultation in a puppy.

**Figure 5:**
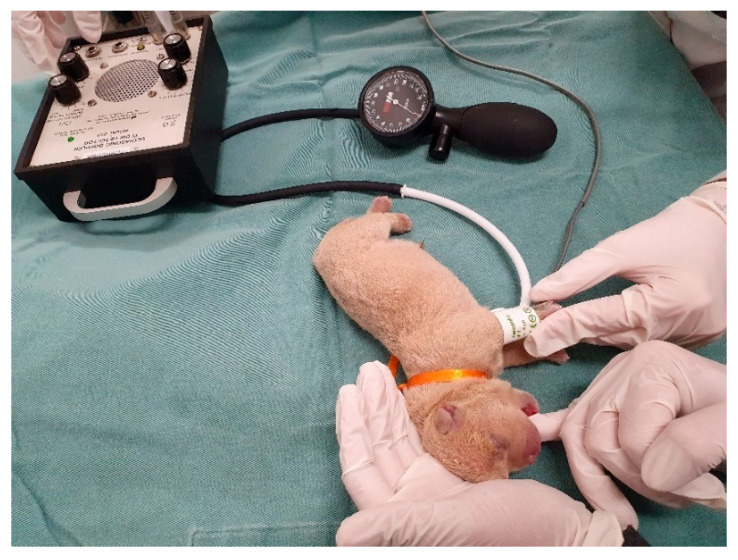
Measurement of blood pressure in a neonatal puppy.

Heart rate can be assessed with the aid of a neonatal stethoscope (Figure 4) or vascular Doppler (Video S1) on the left side of the thorax or by visualizing the beats in the left thoracic region (visualization of precordial shock). Sometimes, neonates are vocalizing at the time of assessment, which interferes with auscultation; stimulating the sucking reflex by inserting a finger into the newborn’s mouth stops vocalizing, and the heartbeat becomes more audible (Figure 4). The canine and feline neonatal heart rates are described in Table 3. Measuring blood pressure in neonatal puppies (Figure 5) and kittens can be challenging in clinical practice because of the small size of the patients and restricted cuff size options. Therefore, this assessment is often limited to older neonates or those with a larger thoracic limb. For adequate blood pressure assessment, the cuff width must be 40% of the size of the thoracic limb [22,23]. However, in the first week of life, the thoracic limb of a feline or canine newborn (from a small breed) measures an average of 4 cm in diameter; on the other hand, the smallest veterinary cuff (n°1) is 2.5 cm wide. Therefore, there is a need for smaller veterinary cuffs for adequate neonatal assessment.

The control of the newborn’s respiratory function develops before birth, maturing during the postnatal period. Canine and feline neonates have structurally immature lungs, less expansion capacity of the thorax wall, immature carotid chemoreceptors, and high metabolism, which is why they are more predisposed to hypoxia. Volume and minute ventilation are lower, but the respiratory rate (Table 3) is greater than in adults [1,16,24,25]. Neonatal kittens have greater heart and respiratory rates than puppies. It is possible that the feline species present greater maturity of the cardiorespiratory system [16,26]. However, additional studies are needed to examine cardiorespiratory maturity in newborn cats compared to dogs. The respiratory rate is assessed by observing chest expansion movements, leaving the newborn on the assessment table (Video S2).

**Figure 6:**
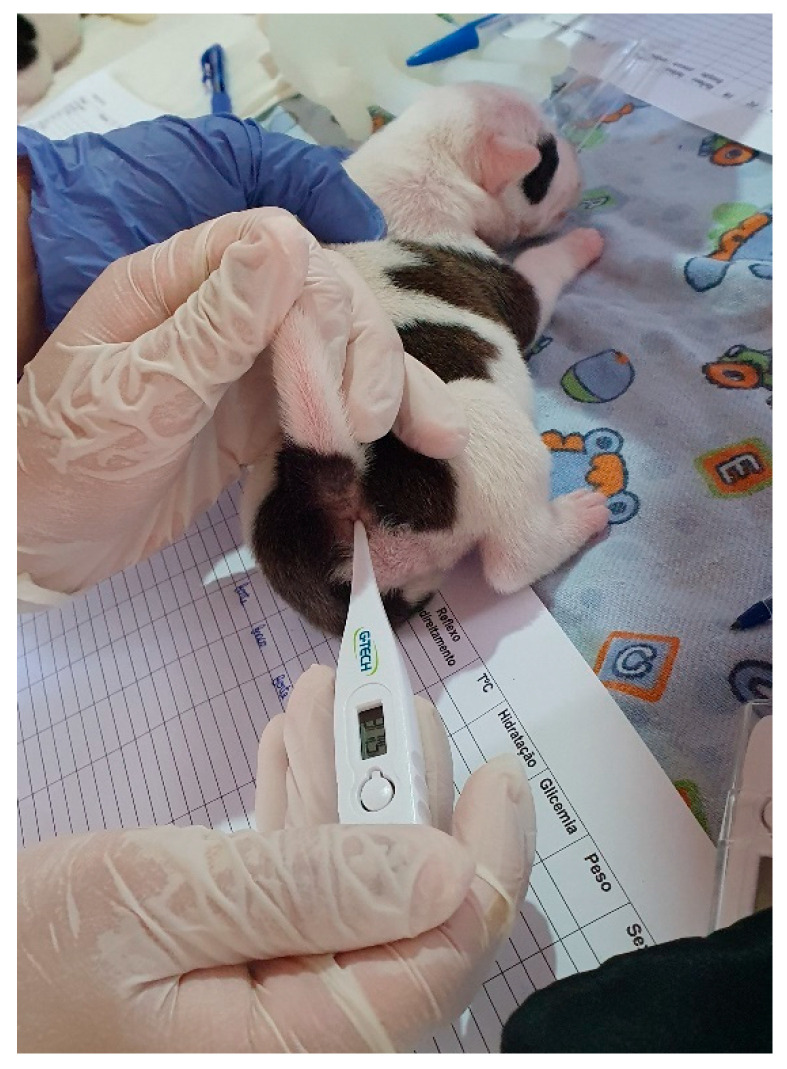
Assessment of rectal temperature in a puppy.

**Figure 7:**
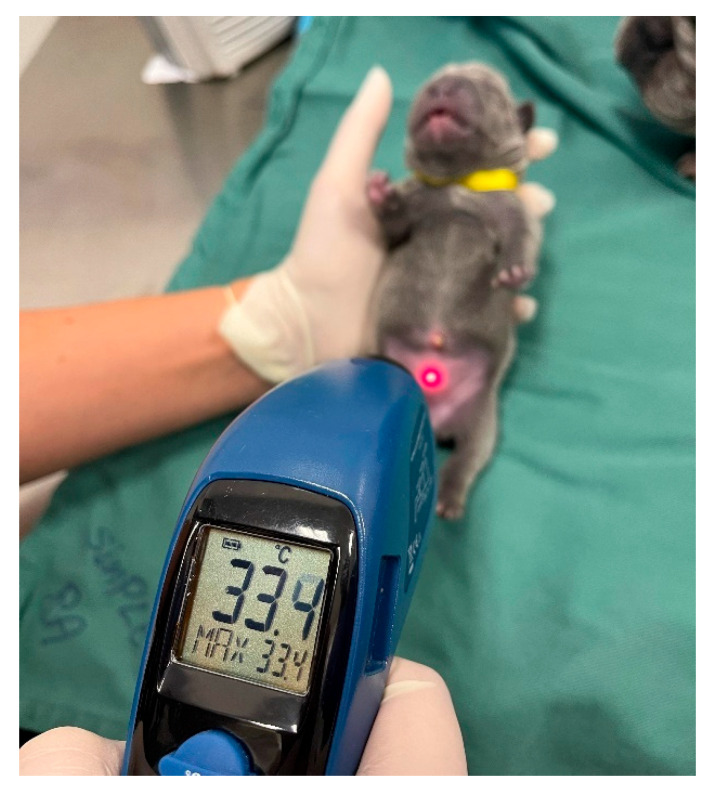
Assessment of temperature with an infrared thermometer.

Due to hypothalamic immaturity, the temperature of neonatal puppies and kittens is lower than that of adult animals (Table 3), gradually increasing over time and approaching adult parameters in the fourth and sixth weeks of life in puppies and kittens, respectively, when complete maturity of the thermoregulatory system occurs [1]. The neonate’s temperature can be assessed rectally (using a digital thermometer with a soft tip) (Figure 6) or using an infrared thermometer pointed to the neonate’s abdomen (Figure 7).

#### 3.3.2. At Birth: Modified Apgar Score

The Apgar score is a simple and reliable method of systematic assessment of vitality, neurological clinical depression, and viability at the time of birth. The principle of this score is to evaluate the immediate health of the newborn and provide quick assistance to critical patients or even evaluate the effectiveness of the provided assistance. The method was developed by Doctor Virginia Apgar after three years of observing newborn babies at the Sloane Hospital for Women, New York [14,27,28]. In veterinary medicine, the score has been described with modifications for several species, including dogs and cats [14,16,21].

Despite the evolution of studies with puppies and neonatal kittens, there is a discrepancy in published studies between these species, with fewer data available on kittens. For example, the modified Apgar score for dogs and its ability to predict survival was first proposed and described in the 2000s [14], after which several studies have been published over the years [13,15,29,30,31,32]. However, in kittens, the first proposed modified Apgar score was published in the 2020s [16], with a delay of almost 20 years of studies and important parameters for feline species. This resulted in dog parameters being used for cats in clinical practice for years, erroneously, since there are clear differences in parameters between these species [16]. Therefore, regarding the use of the Apgar score, differences between breeds and assessment of survival prognosis, more studies should be carried out in kittens.

The modified Apgar score for puppies and kittens (Table 4 and Table 5, respectively) is based on the evaluation of mucous membrane color, heart rate, respiratory rate, muscle tone, and reflex irritability (Videos S3 and S4), which are assigned grades of 0, 1 or 2, according to what was presented by the newborn, with the sum of the grades ranging from 0 to 10 [14,16,21].

Mucous color is assessed by visualizing the oral mucosa. Heart rate and respiratory rate can be measured with the aid of a stethoscope on the left side of the chest and by observing the expansion of the chest wall, respectively, as previously described. Muscle tone is determined with the newborn in the supine position on the palm of the hand, observing active movements of the limbs and body. The irritability reflex is assessed after a painful stimulus caused by pressing the interdigital space [14,16,21,33].

The interpretation of the score is evaluated according to the species and differences related to the body size characteristics of the breed (Table 6) [34]. Normal viability scores/without respiratory distress are considered ideal, with newborns being healthy and in clinical conditions favorable for survival. Moderate viability/moderate respiratory distress scores may indicate a possible need for resuscitation and neonatal monitoring, whereas neonates with poor viability/severe respiratory distress scores indicate a risk of imminent death. The vast majority of these patients experience prolonged asphyxia in the uterus and consequent prominent hypoxia at birth, indicating an emergency need for resuscitation [6,14,16,21,34].

Neonates are susceptible to hypoxemia because of the need for high oxygen metabolism, lung immaturity, and the immaturity of carotid chemoreceptors [1]. Although they may present physiological hypoxemia shortly after birth, resulting in hypercapnia and mixed acidosis, this is transient [13,21]. However, prolonged and dystocic births can lead to pronounced asphyxia and severe hypoxia in newborns, leading to failure in respiratory adaptation and the need for intervention, with the Apgar score being an essential tool in the diagnosis and monitoring of these hypoxemic patients [14,21,33].

The modified Apgar score is a predictor of mortality; scores below 5 in small dogs and below 6 in medium and large dogs are strongly associated with the risk of death in the first 24 h, especially if these patients do not receive adequate assistance [34].

The modified Apgar score must be determined within the first five minutes after birth and repeated after 10 and 60 min to evaluate the clinical evolution of the newborn. It is important to remember that the score should not replace the complete physical examination of the patient in the first hours of life but rather assist in the quick classification of the newborn’s condition at birth [35].

#### 3.3.3. Neonatal Reflexes

The main neurological reflexes (sucking, rooting response, and vestibular righting) (Videos S5 and S6) are present at birth and can be used to assess neonatal vitality. Healthy newborns must have strong reflexes, which allow breastfeeding and adequate development. On the other hand, any neonatal condition/disease that induces apathy and clinical depression culminates in reduced reflexes (weak vitality), failure to breastfeed, and progression to the neonatal triad (hypoglycemia, hypothermia, dehydration). In this way, the neonatal reflex score can be evaluated in cases where a neonatal alteration is suspected, favoring either early diagnosis and intervention or monitoring the clinical improvement of the newborn after the institution of treatment.

The sucking reflex must be assessed by inserting the tip of the examiner’s finger into the newborn’s mouth and observing the strength of the suction, which must be strong, presenting a vacuum during the evaluation. The rooting response reflex can be performed by placing the examiner’s hand close to the newborn’s face, who must immediately search for the mammary glands. The righting reflex is stimulated by placing the newborn in the supine position under a soft, heated surface. The expected response of this reflex corresponds to the straightening of the newborn’s body, with a quick return to the prone position. Each reflex receives a score of 0, 1, or 2 (absent, weak, or strong response, respectively) (Table 7), and the sum is used to indicate neonatal vitality. The score is evaluated as follows: 0–2, weak vitality; 3–4, moderate vitality; and 5–6, normal vitality [21,36].

In conjunction with the rooting response reflex, the newborn’s march reflex can be assessed (Video S7); however, due to its immaturity, the newborn moves by dragging its thorax and abdomen through swimming movements with its limbs. The objective of this reflex is to observe neonatal vigor in moving (crawling) in search of the mammary glands, determining its vitality. This reflex is present from birth, but the ability to support the body with the pelvic limbs occurs later, approximately 14 to 16 days of age, when the newborn begins to walk. Other neurological assessment reflexes included the magnum and landau. The magnum reflex is characterized by the rotation of the newborn’s neck to the side, with an expected extension of the ipsilateral thoracic and pelvic limbs, rotation of the head, and flexion of the contralateral limbs. The response is usually most evident in the thoracic limbs. The landau reflex (seal posture) is assessed by supporting the newborn in the prone position on the palm of the hand. The expected response is opisthotonus and extension of the pelvic limbs and tail. The magnum and landau reflexes are present until the third week of age [2,36], and the persistence of these reflexes denotes delayed neonatal neurological development.

#### 3.3.4. Muscle Tone

Muscle tone, as previously described in the Apgar score, can be assessed with the newborn in the supine position on the palm of the hand, observing the movements of the limbs and body contortion (Video S8). The objective is to determine the vitality of the newborn, which may be absent, weak, or strong (see Table 4) [16,21]. A strong tone is essential for adequate movement and breastfeeding of the newborn. However, the movements of newborns are uncoordinated, demonstrating cerebellar immaturity. Tone can also be used for neurological assessment: by elevating the neonate by the mastoid region, a flexor posture of the body or pelvic limbs is observed until four to five days of age, which is later replaced by an extensor posture of the body or pelvic limbs, which lasts until the third or fourth week of age, followed by normotonia [36].

#### 3.3.5. Assessment of the Neonatal Triad

During the neonatal period, puppies and kittens present immaturity in several organic systems, possessing unique characteristics that completely differ from those of adult animals. One of the main consequences of physiological immaturity is the greater risk of manifestation of the neonatal triad (hypothermia, hypoglycemia, and dehydration).

The neonatal triad is the most common clinical manifestation of any condition or change in the newborn. It is important to highlight that any neonatal disease can cause depression, apathy, a reduced sucking reflex, and a consequent reduction in milk intake, which can lead to the manifestation of this condition. Furthermore, the triad can occur due to management errors and maternal changes, such as agalactia, hypogalactia, or failure of maternal instinct. Assessment of the triad is essential at any neonatal appointment for puppies and kittens.

##### Hypothermia

Several physiological aspects are related to the predisposition of newborns to develop hypothermia, such as hypothalamic immaturity, reduced adipose tissue, poorly vasoconstriction mechanisms, inability of the tremor reflex in the first days of life, and a large surface/body mass ratio [1,2,37]. Therefore, newborns are considered ectothermic animals, requiring maternal or environmental heating to maintain a stable body temperature and active metabolism.

Moderate hypothermia can reduce metabolism, leading to lethargy, inappetence, and decreased neonatal reflexes. The newborn still tries to breastfeed, but the milk may not be properly digested due to reduced motility of the gastrointestinal tract (paralytic ileus), which can cause regurgitation, aspiration pneumonia, gas production, and gastrointestinal dilation [1,38]. For these reasons, hypothermic neonates should not be fed.

In severe hypothermia, neonatal clinical depression occurs, and newborns become extremely lethargic, interrupting attempts to breastfeed. There is a decrease in cardiorespiratory function (bradycardia and bradypnea), resulting in a risk of tissue hypoxia, acidosis, and death [38].

To diagnose hypothermia, the newborn’s temperature must be assessed rectally (using a digital thermometer) (Figure 5) or using an infrared thermometer (Figure 6), as previously described. The newborn’s ideal body temperature varies with age (Table 3).

##### Hypoglycemia

Some physiological characteristics make neonates particularly susceptible to hypoglycemia. Due to liver immaturity, puppies and kittens are born with limited glycogen reserves and minimal capacity for gluconeogenesis. In puppies and kittens that are not breastfeeding, blood glucose levels can decline quickly as the ability to maintain normoglycemia during fasting is reduced. Liver reserves are completely depleted within 24 h; however, a rapid decline in glycemia may occur before this period in fragile, sick, premature, or low-birth-weight neonates [1,39].

Hypoglycemic newborns may manifest crying, weakness, apathy, decreased or absent sucking reflex, and interruption of breastfeeding, worsening their condition. Severe hypoglycemia can lead to bradycardia, seizures, coma, and death. Bradycardia occurs due to reduced metabolism and glucose supply to the myocardium. As the treatment is carried out and the blood glucose increases, the heart rate increases concomitantly [39].

**Figure 8:**
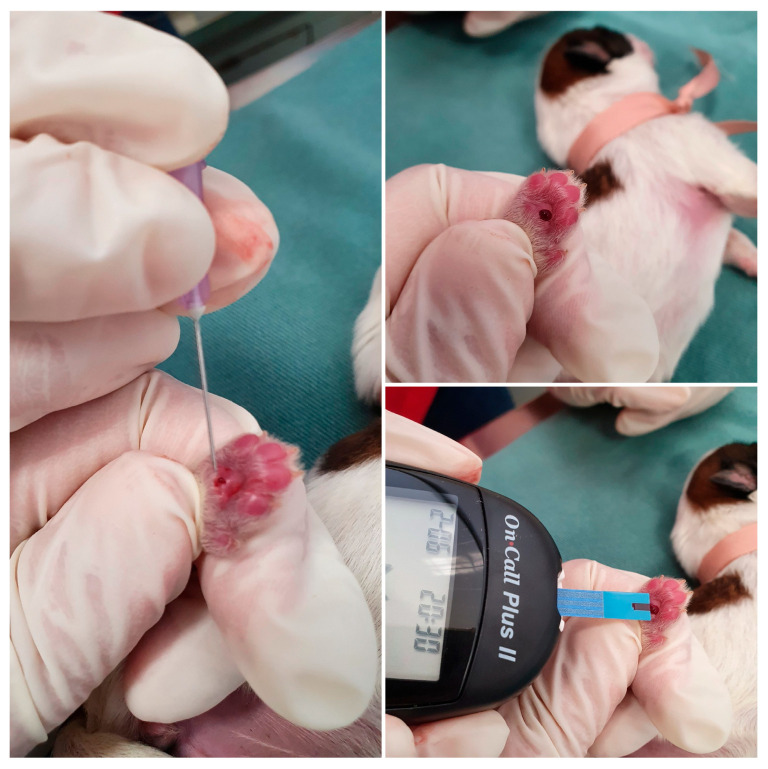
Blood collection from the neonatal puppy digital pad and blood glucose measurement.

Blood glucose should be measured with a portable glucometer, using a 24 or 26-gauge needle to collect a blood sample from the newborn’s digital pad (Figure 8), from the inside of the ear, or by collecting blood from the jugular vein.

The glycemic reference value in puppies and neonatal kittens is still controversial. In dogs, an old study demonstrated that concentrations such as 40 mg/dL [40] shortly after birth were considered normal. A more recent study reported an average concentration of 66 mg/dL in the umbilical cord [41]. However, another recent study demonstrated that at birth, the glycemia of neonatal dogs is related to maternal glycemia (approximately 90%) [42], and this cannot be considered a standard of normality for the species. Another study demonstrated that a glucose concentration <92 mg/dL during the first 24 hours of life is correlated with a greater risk of death during the first 21 days of life [43], indicating that concentrations lower than these are not considered normal. For example, a study demonstrated that the average blood glucose levels in newborn puppies aged one, four, and seven days were 106, 114, and 129 mg/dL, respectively [12]. With respect to glycemia during the first four weeks of age, one study reported a mean glycemic level of 125 mg/dL (range 106–182 mg/dL) in 349 healthy neonatal dogs [44]. Thus, neonatal normoglycemia in dogs can be considered to be between 90 and 200 mg/dL [39]. A glucose concentration <90 mg/dL indicates hypoglycemia [39,43]. Neonatal hypoglycemia can be characterized as mild (<90 to 70 mg/dL), moderate (<70 mg/dL to 40 mg/dL), or severe (<40 mg/dL) [39].

In neonatal kittens, one study demonstrated that the mean blood glucose at birth (eutocic parturition) was 68 mg/dL (ranging from 26 to 151 mg/dL) [16], but possibly, in the same way as in puppies, glycemia at birth may be related to maternal glycemia. Authors suggest that a blood glucose concentration <50 mg/dL in kittens up to two weeks after birth is abnormal, especially when accompanied by clinical signs [37,45]. In the literature, a range of 75 to 154 mg/dL on the second day after birth is considered normal [45]. More studies are needed to evaluate the average blood glucose levels of kittens over time during the neonatal period.

##### Dehydration

Newborn puppies and kittens are more susceptible to dehydration than adult animals, mainly due to renal immaturity, with a lower water conservation capacity. The puppy neonatal kidney is functionally characterized by a low glomerular filtration rate, renal plasma flow, filtration fraction, depressed reabsorption of aminoacids and phosphate, and low concentrating ability [46,47,48]. In the literature, information concerning the renal physiology of neonatal kittens is lacking. Furthermore, other physiological characteristics, such as a higher concentration of body water, a large surface area/body mass ratio, and greater loss of fluids due to skin immaturity or a thin layer of keratin, increase the predisposition of newborns to dehydration [1,2]. Dehydrated puppies can progress to hypovolemia, hypotension, shock, and death.

**Figure 9:**
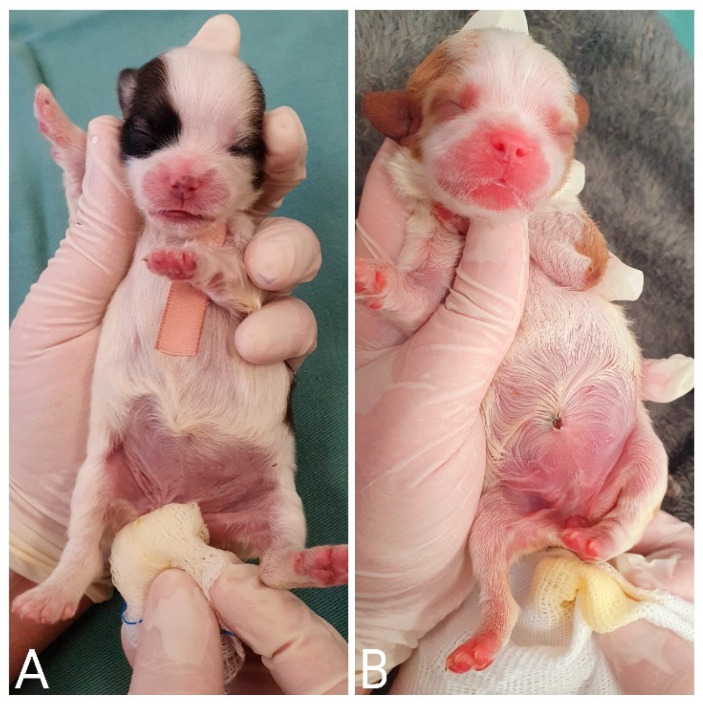
Differences in urine color. (**A**) Normohydrated neonate whose urine color was close to transparent. (**B**) Dehydration of the neonate (dark yellow urine).

It can be challenging to assess hydration status in canine and feline neonates. Due to the physiological immaturity of the skin, lower subcutaneous fat, and higher concentration of body water, skin turgor is not developed as in adults and is not a reliable assessment in neonates. The color of mucous membranes can be used to assess hydration; the dark red color is indicative of dehydration in newborns [49,50]. Neonatal dehydration can be assessed subjectively by the urine color, which in newborns is very diluted with no visible color [6,10,50]. Shades of yellow suggest dehydration; the darker the urine is, the more dehydrated the newborn is. A gentle manipulation of the genitalia with a damp cotton pad stimulates urination and evaluation of urine color (Figure 9). Furthermore, urinary density can be evaluated using a refractometer, where a value > 1.017 is considered an indication of dehydration. As the kidney is not able to concentrate urine effectively, the specific gravity of urine gives results when there is already possible severe dehydration [1,2,50].

Other subjective methods of assessing dehydration include laboratory parameters such as hematocrit and total protein. The hemoconcentration is an indication of dehydration. Hemograms must be evaluated according to the normal parameters of the neonatal period.

After evaluation of temperature, blood glucose, and hydration, a physical examination should proceed. However, if the neonate is diagnosed with a triad, it is essential first to stabilize the newborn and then continue the diagnostic investigation.

#### 3.3.6. General Physical Examination

**Figure 10:**
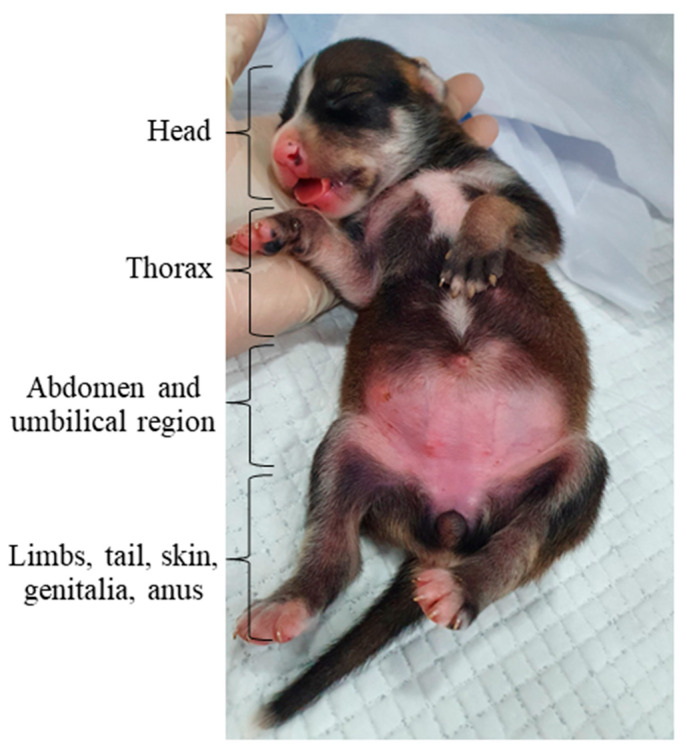
Regions to be evaluated in the systematic physical examination of the newborn.

To identify puppies and kitten neonates at risk, each part of the newborn’s body must be carefully analyzed, such as the head, chest, abdomen, limbs, tail, skin, anus, and genitals (Figure 10), in search of possible changes.

##### Head

In the head region, we must evaluate the shape, size, symmetry, and presence of the fontanelle, ears, eyelids, eyes, oral and nasal cavity, and lymph nodes since each region can demonstrate neonatal changes that often go unnoticed.

**Figure 11:**
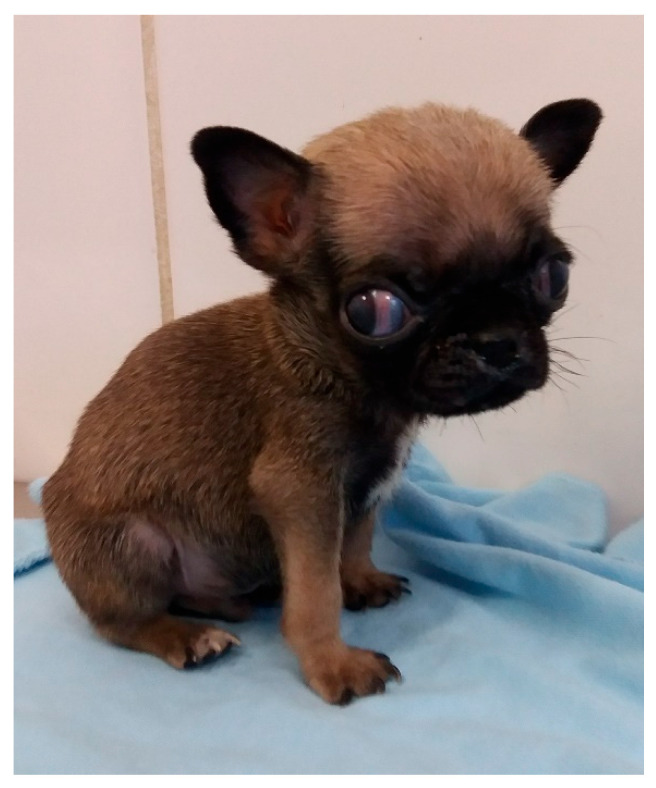
Macrocephaly and strabismus in a neonatal puppy with hydrocephalus.

**Figure 12:**
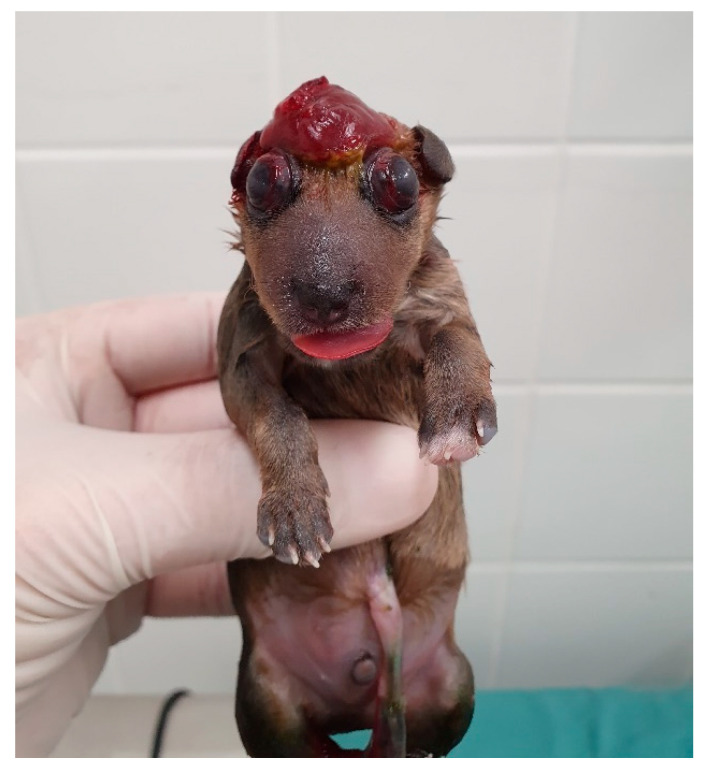
Cranioschisis, exencephaly, and eyelid coloboma in a neonatal puppy.

**Figure 13:**
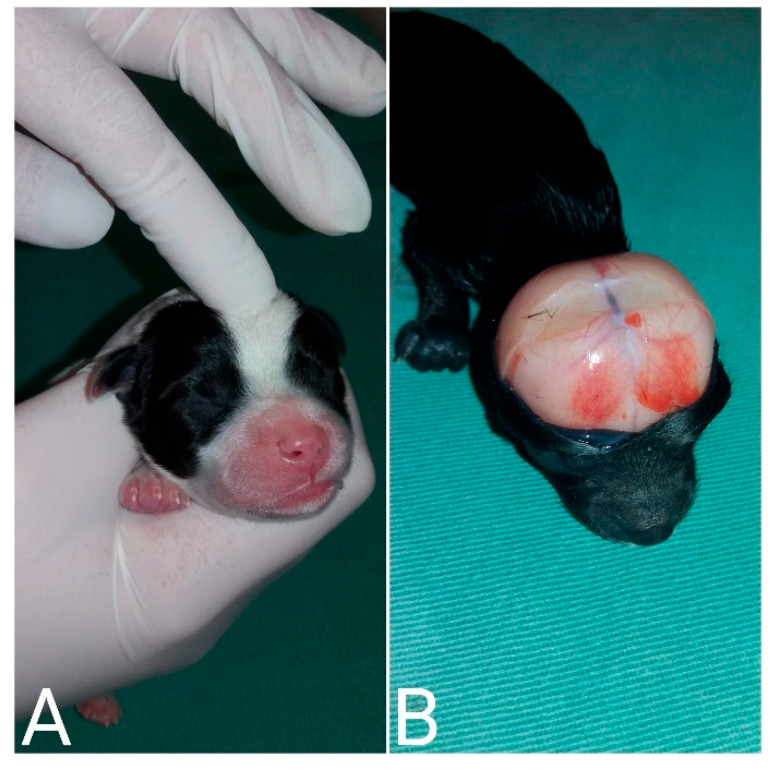
(**A**) Palpation of the skull to identify an open fontanelle. (**B**) Neonatal necropsy demonstrating the presence of a fontanelle (nonclosing of the bone fissures) in a puppy with hydrocephalus.

**Figure 14:**
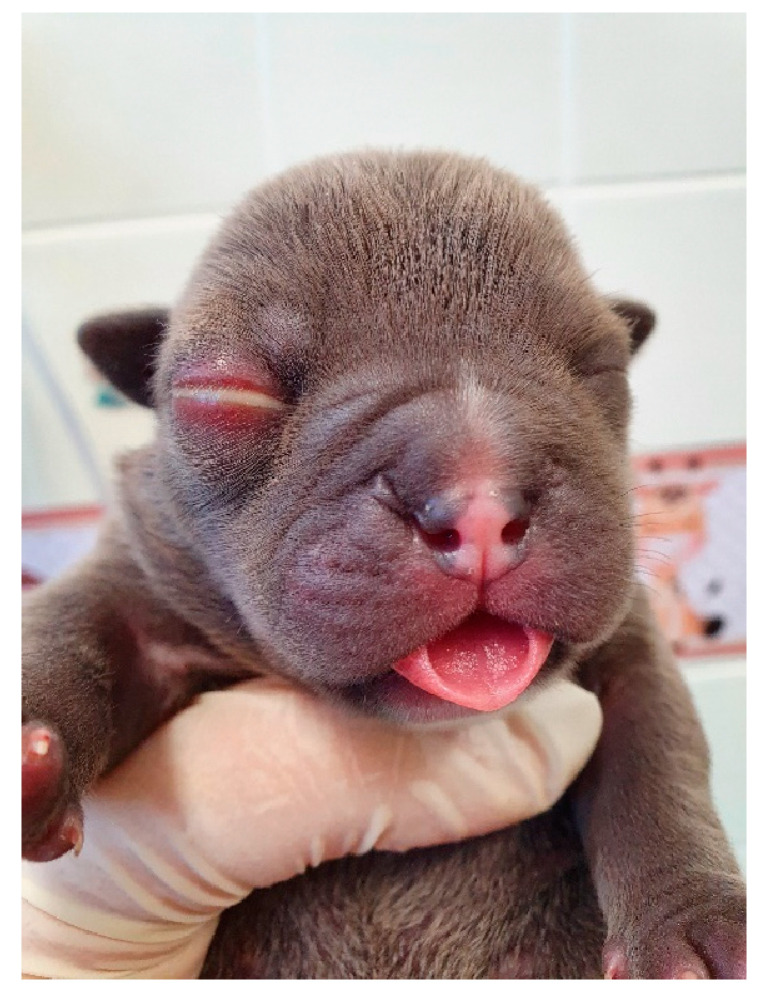
Neonatal ophthalmia in a puppy.

**Figure 15:**
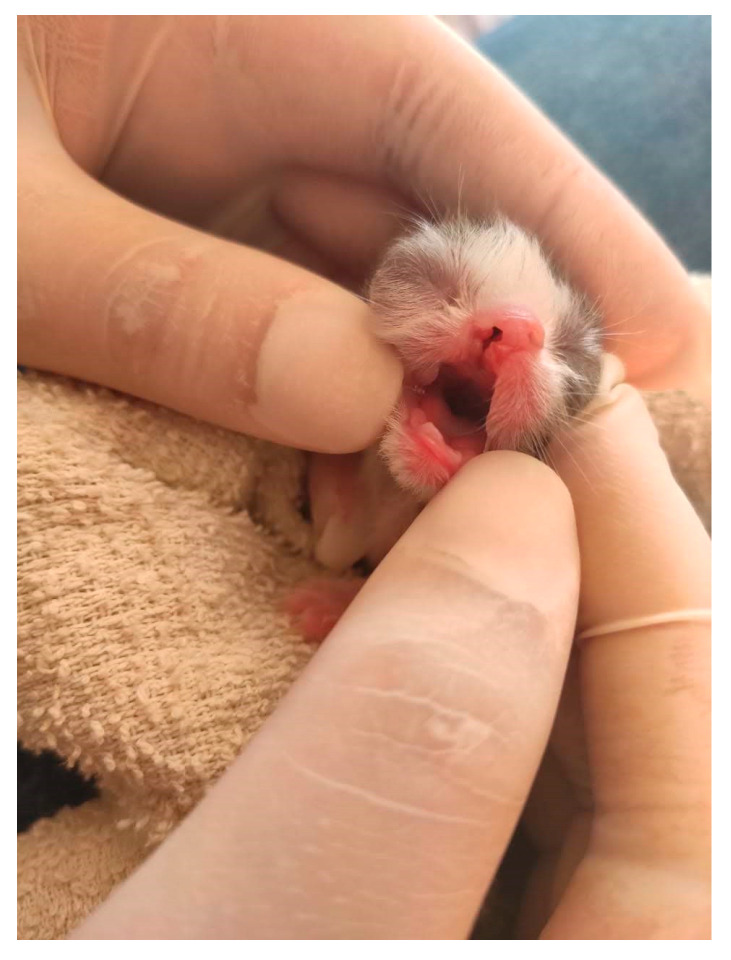
The cleft lip in a neonatal kitten.

**Figure 16:**
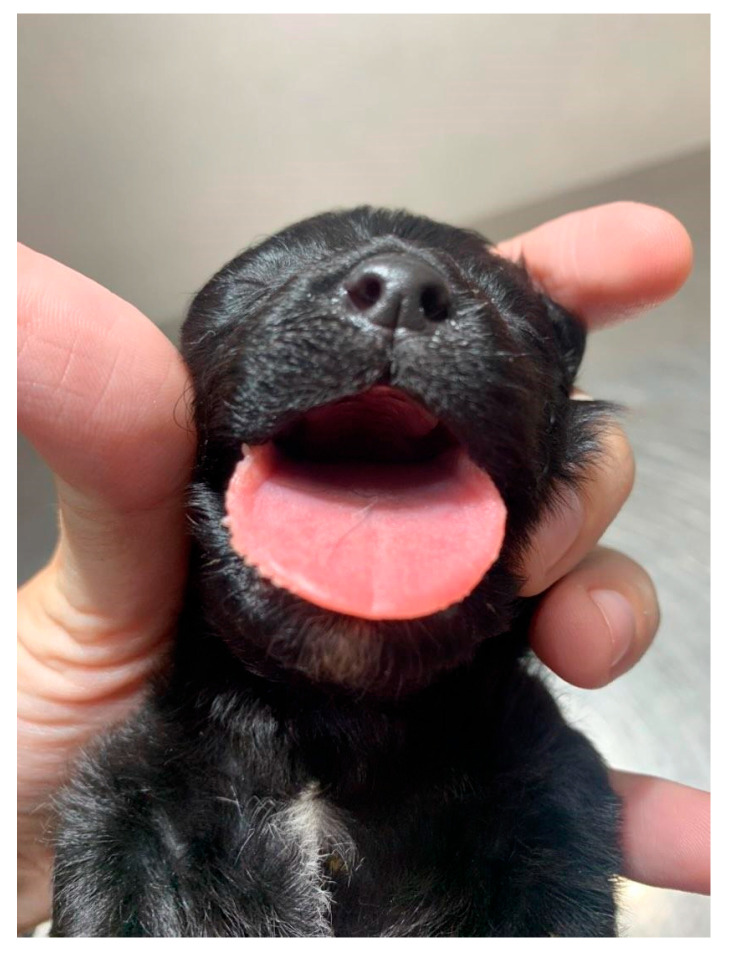
Macroglossia in a neonatal puppy.

**Figure 17:**
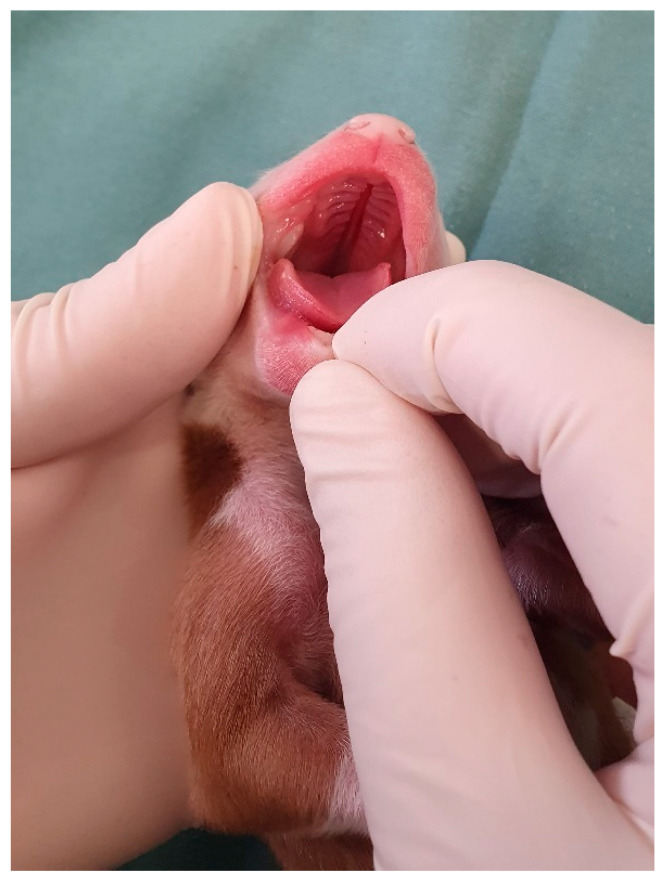
Cleft palate in a neonatal puppy.

**Figure 18:**
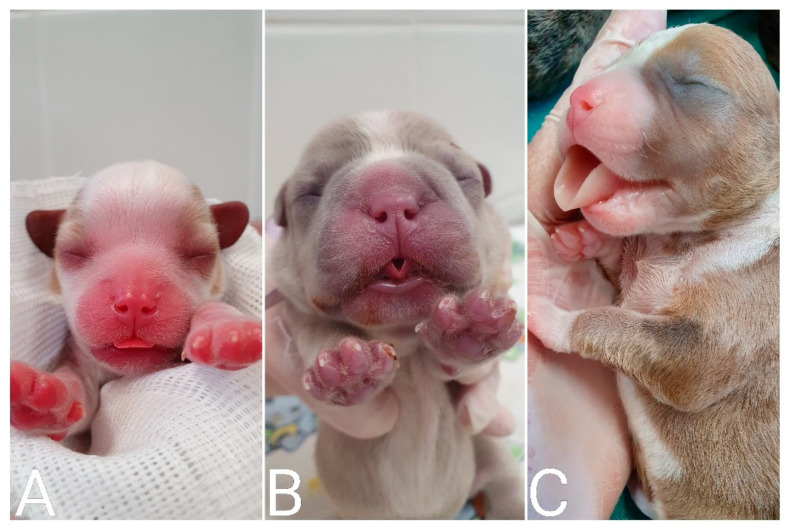
Coloration of mucous membranes in newborn puppies. (**A**) Intense pink color; (**B**) cyanotic; and (**C**) pale.

- Shape/size/symmetry: changes such as macrocephaly can be a clinical sign of hydrocephalus (Figure 11), a common malformation in neonatal puppies and kittens, especially in brachycephalic breeds. Hydrocephalus can cause neurological dysfunction, such as seizures, which in newborns can be identified by generalized muscle rigidity (most commonly observed) (Video S9) or pedaling movements (Video S10). Cranioschisis and exencephaly (Figure 12) are also frequently observed malformations;
- Fontanelle: the skull of the newborn must be palpated to assess the presence of a fontanelle (nonclosure of bone fissures) (Figure 13). Some newborns may have an open fontanel at birth; however, this should close within the first few days of birth. A persistent fontanelle may also be a clinical sign of hydrocephalus and should be investigated;
- Ear and ear canal: neonates are born with a closed ear canal, which should open on average between 12 and 17 days of age [2]. One should always evaluate the shape, insertion, size, and presence of changes in the ear canal and ear, such as otitis and anotia (absence of one or both ears);
- Eyelids and eyes: neonates are born with their eyelids closed, beginning opening on average between eight to 12 days in kittens and 12 to 15 days in puppies [2,10]. The size and symmetry of the eyelids and eyeballs must be observed. A common condition in newborns is neonatal ophthalmia, characterized by infection of the conjunctiva and cornea before opening the eyelids, where there is an increase in volume in the ocular region due to the accumulation of purulent secretions (Figure 14). In some cases, this increase in volume may be subtle; therefore, it is important to carefully evaluate each newborn. Other frequent disorders in neonates are ventrolateral strabismus (common in hydrocephalus) (Figure 11), conjunctivitis, corneal ulcers, and malformations, such as palpebral coloboma (absence of eyelids) (Figure 12), microphthalmia and anophthalmia [51]. The pupillary light reflex begins between 10 and 16 days of age in puppies and between 5 and 14 days of age in kittens. The eyelid reflex begins between two and four days of age in puppies and between one and three days in kittens. The threat response starts between 10 days and 4 weeks in puppies and between 7 days and 4 weeks in kittens [2].
- Oral cavity: abnormalities in the lip (cleft lip) (Figure 15), position and size of the tongue (such as macroglossia) (Figure 16), and changes in the hard and/or soft palate (cleft palate) (Figure 17) can be diagnosed [51,52]. Additionally, the color of the muzzle or mucous membranes must be evaluated, which in healthy newborns must be intense pink; other colors may indicate changes, such as cyanotic (e.g., perinatal asphyxia/hypoxia, heart disease, neonatal triad, sepsis), pale (e.g., anemia, hypotension, heart disease, septic shock, feline neonatal isoerythrolysis) (Figure 18), hyperemic (e.g., systemic bacterial infections/sepsis, dehydration), and jaundice (e.g., hemolytic anemia, feline neonatal isoerythrolysis, infections, liver changes). It is also important to evaluate neonatal dentition. Puppies and kittens are born edentulous, with tooth eruption starting from the third week of age. The dentition can be used to estimate the age of newborns.
- Nasal cavity: observe the presence of nasal secretions, epistaxis, nostril stenosis (brachycephalic syndrome), obstructions, and cleft lip, which can extend to the nostril unilaterally or bilaterally.
- Lymph nodes: depending on the size/port of the newborn, the submandibular and popliteal lymph nodes are palpable. Enlargement of submandibular lymph nodes is common in juvenile cellulitis and submandibular abscesses. Occasionally, the inguinal lymph nodes can be visualized when they are enlarged.

##### Thorax

Thoracic evaluation is based on inspection of the breathing pattern, observation of asymmetries and malformations of the spine and rib cage, assessment of heart and respiratory rates, and cardiopulmonary auscultation to detect cardiac, pulmonary, esophageal, and tracheal disorders.

**Figure 19:**
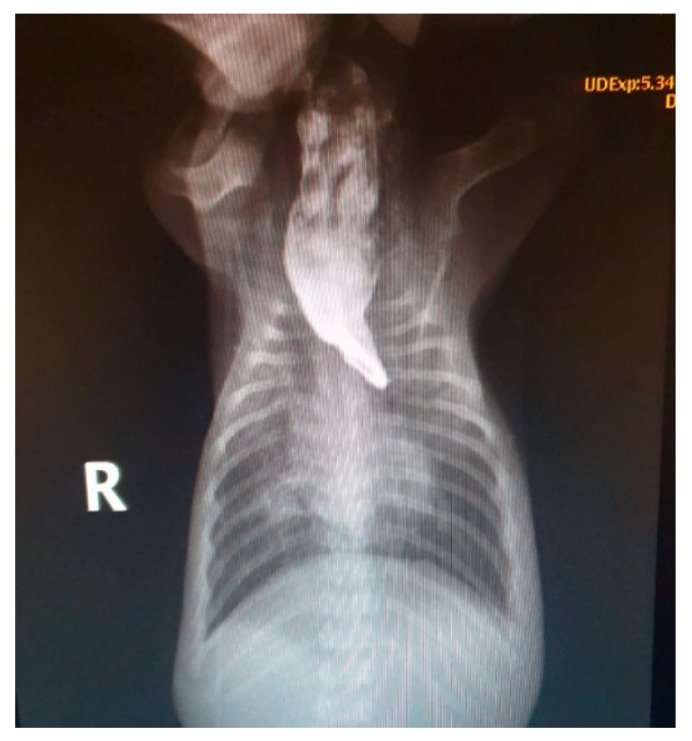
Contrast thorax X-ray demonstrating congenital megaesophagus secondary to the persistence of the right aortic arch in a neonatal puppy.

**Figure 20:**
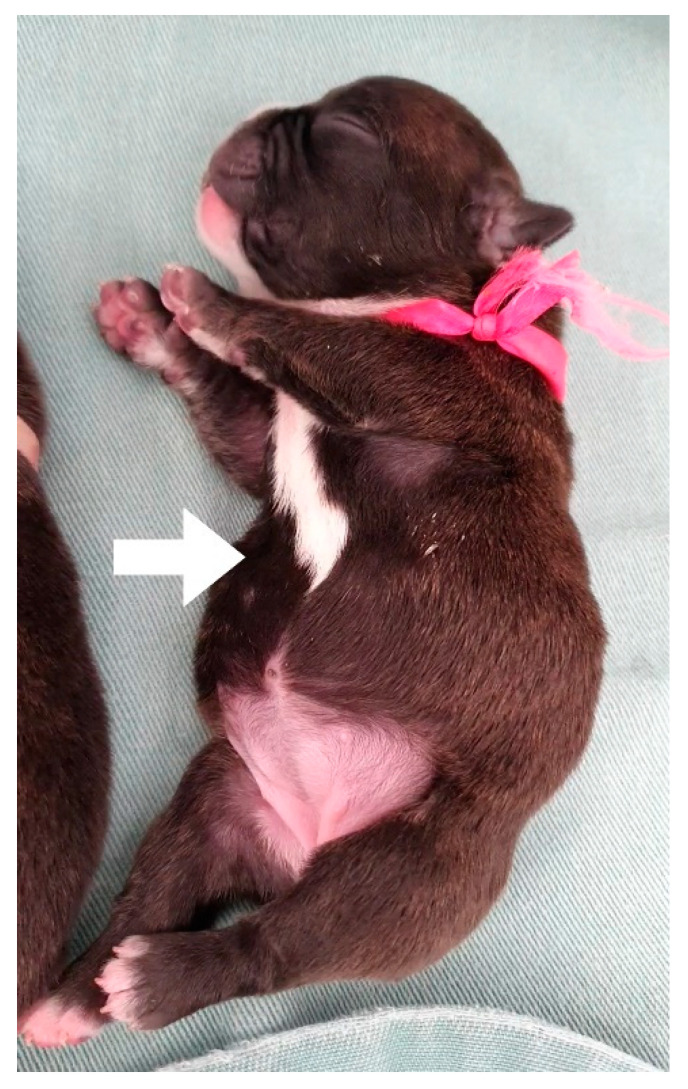
Pectus excavatum in a neonatal puppy. Ventrodorsal narrowing is observed in the sternal region (arrow).

- Heart rate: As previously described, the heart rate of neonatal puppies should be between 200 and 260 bpm and that of kittens should be between 200 and 280 bpm. Bradycardia can be observed in several neonatal changes and conditions, including perinatal asphyxia/hypoxia, hypothermia, hypoglycemia, heart disease, systemic bacterial infections/sepsis, and septic shock [10,13,21].
- Respiratory rate and pattern: the presence of eupnea with a respiratory rate between 15 and 40 mpm in puppies and 40 and 180 mpm in kittens should be assessed. Neonates always breathe with their oral cavity closed; observing movements of the mouth opening to breathe indicates respiratory distress. Changes in respiratory patterns, such as dyspnea (Video S11), tachypnea, and bradypnea, occur in newborns with cardiorespiratory abnormalities, such as perinatal asphyxia/hypoxia, congenital cardiac anomalies, pulmonary edema, pneumonia, brachycephalic syndrome, prematurity, hypothermia, pectus excavatum, and septic shock [2,13,16].
- Cardiopulmonary auscultation: the presence of abnormal sounds, such as wheezing, crackles, stridor, cardiac arrhythmias, and murmurs, should be assessed.
- Asymmetries and malformations: abnormalities in the spine (e.g., scoliosis), cardiomegaly, tracheal hypoplasia, megaesophagus (Figure 19), lung changes, rib cage deformities (e.g., pectus excavatum (Figure 20), thoracoschisis), diaphragmatic hernia, among others [2,51].

##### Abdomen

Color changes, omphalitis, distension, hematomas, pustules, eviscerations, and malformations must be evaluated in the abdominal region.

**Figure 21:**
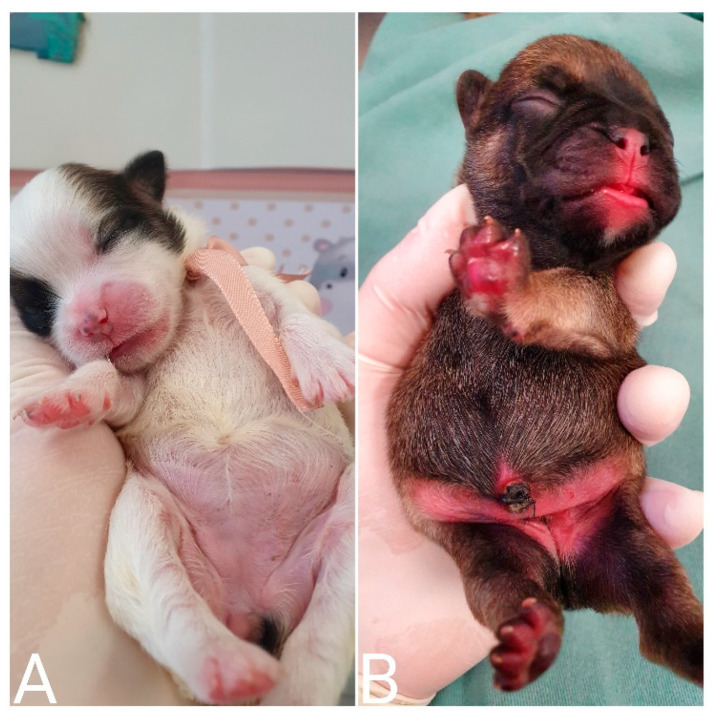
(**A**) Light pink abdominal color in a healthy neonate. (**B**) Abdominal/body erythema in a neonate with systemic bacterial infection.

**Figure 22:**
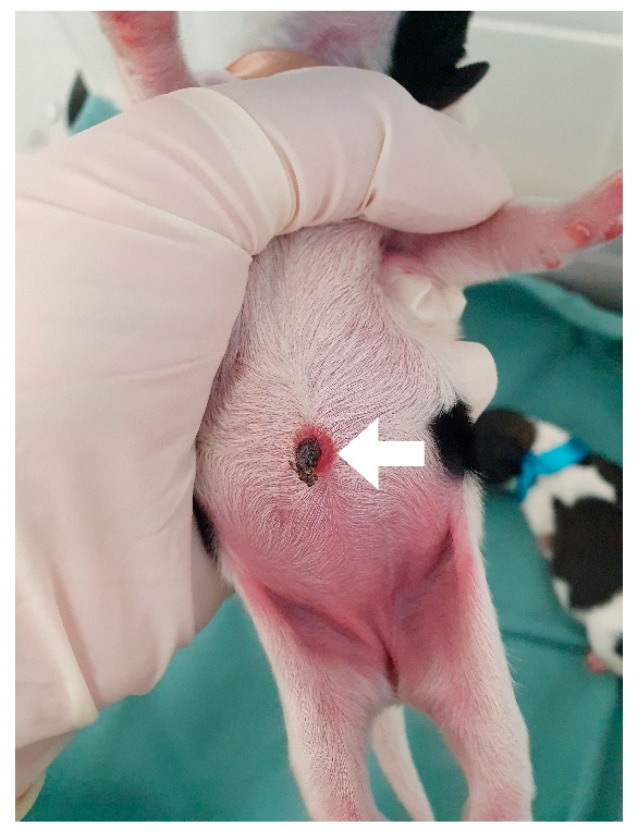
Omphalitis in a neonatal puppy with systemic bacterial infection. A hyperemic halo can be seen in the umbilical stump (arrow).

**Figure 23:**
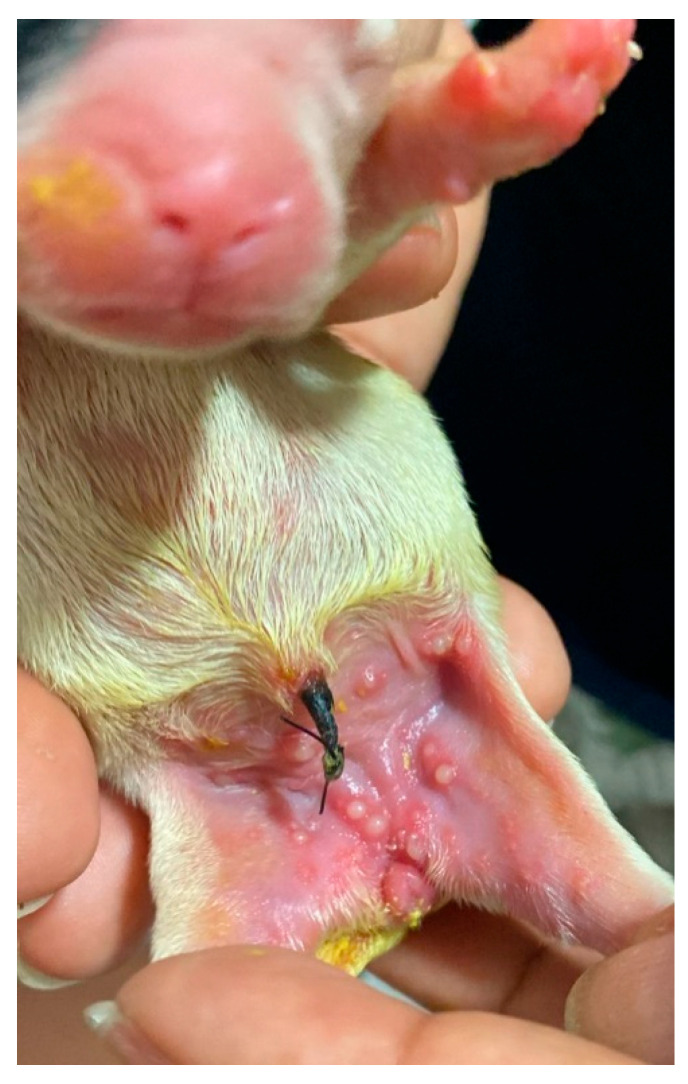
Abdominal pustules in a neonatal puppy that did not ingest colostrum, presenting failure in passive immunity transfer and systemic bacterial infection.

**Figure 24:**
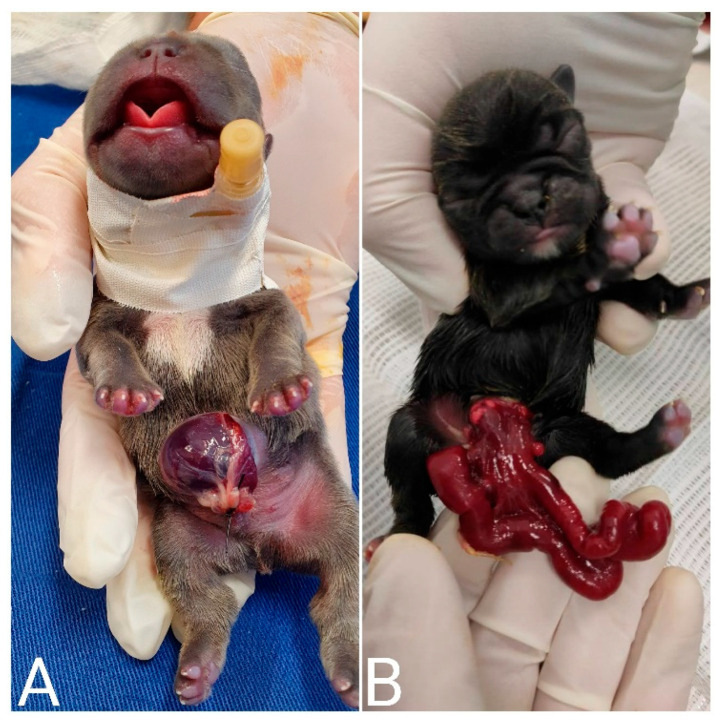
Omphalocele (**A**) and gastroschisis (**B**) in neonatal puppies. Both congenital defects are eviscerations; however, in omphalocele, the viscera are contained within a thin membrane, usually the peritoneum. In gastroschisis, the viscera are exposed.

- Color changes: the abdomen of a healthy newborn has a light pink color. The observation of a reddish (erythematous) color (Figure 21) strongly indicates systemic bacterial infection or neonatal sepsis, which must be investigated immediately with a complete blood count and microbiological culture. Bacterial infections are common in the neonatal period, with sepsis being the main cause of mortality in the first three weeks of life. Abdominal erythema occurs mainly due to vasodilation caused by systemic inflammatory response syndrome [44,53]. Other color changes commonly observed in the abdominal region are cyanotic, pale, and jaundiced, where the possible causes were previously described (see evaluation of the oral cavity);
- Omphalitis is an inflammation of the umbilical region, often caused by infection [2]. It is essential to know that at the time of birth, the umbilical stump has a whitish color that dries out over time and falls off between two and three days of age. Omphalitis can be observed as a hyperemic halo around the umbilical stump (Figure 22), violet coloration of the stump, edema in the region, and the presence of abscess or free purulent secretion. Commonly, the hyperemic halo can present subtly and often go unnoticed, reinforcing the importance of a thorough newborn evaluation in search of this sign. For the most part, a hyperemic halo is a sign of systemic bacterial infection/neonatal sepsis and should be investigated early [44].
- Abdominal distension: increased abdominal volume can be observed in some cases of neonatal changes and diseases, such as the presence of gas in the stomach or intestine, ascites, constipation/obstipation and consequent accumulation of feces, verminosis, and visceromegaly, among others. Abdominal palpation and imaging tests, such as ultrasound and abdominal radiography, can help to determine the underlying cause.
- Hematomas: the presence of abdominal hematomas often indicates hemostatic disorders, which can occur in neonatal sepsis and other infectious processes, liver diseases, neonatal hemorrhagic syndrome, toxic processes, and congenital changes, such as hemophilia and von Willebrand disease.
- Pustules: the presence of abdominal pustules (Figure 23) is commonly observed in infectious processes, mainly in bacterial skin infections (impetigo). In newborns, pustular dermatitis is often associated with immunodeficiency, especially in puppies and kittens that have not ingested colostrum, being often associated with systemic infection.
- Eviscerations and malformations: eviscerations can be observed in trauma (umbilical cord avulsion by the mother) and congenital defects, such as gastroschisis and omphalocele (Figure 24), which require immediate correction surgery. Other malformations may include segmental aplasia of the intestine, hernias, urinary system defects, and portosystemic shunts [51].

##### Genitalia and Anus

The evaluation of the genitalia and anus is based on the observation of urine and feces (color, appearance, volume, consistency) and the presence of malformations.

**Figure 25:**
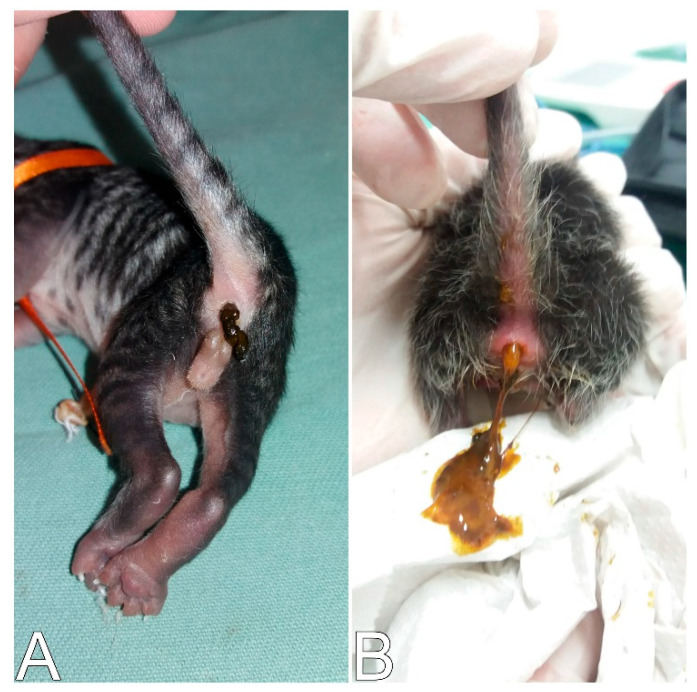
(**A**) Meconium consistent in a healthy neonatal kitten. (**B**) Meconium diarrhea in a neonatal kitten shortly after birth resulting from neonatal sepsis acquired in utero.

**Figure 26:**
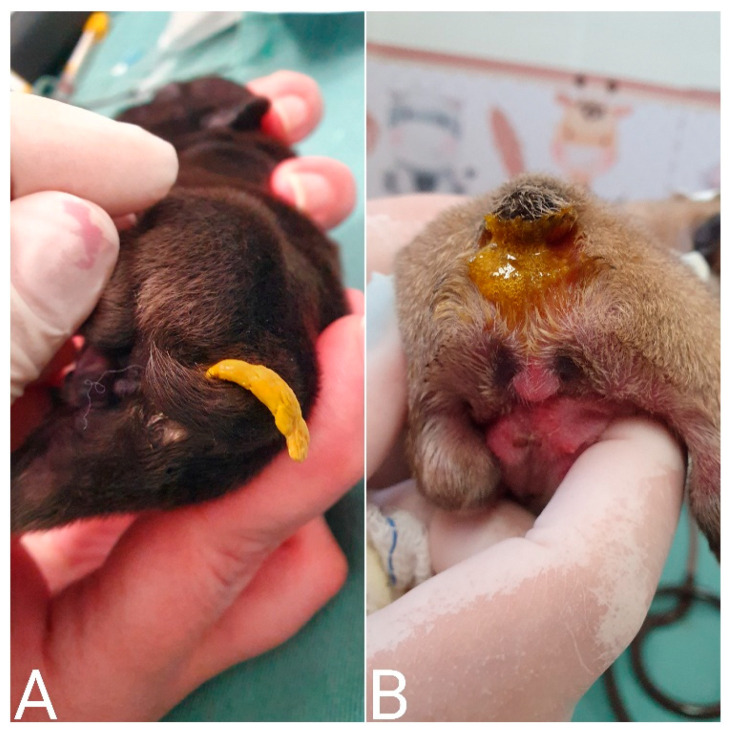
(**A**) Consistent feces in a healthy neonatal puppy. (**B**) Diarrhea in a puppy resulting from neonatal sepsis.

- Genital and urine assessment: to observe urine, urination stimulation must be carried out with gentle massage of the genitalia with the help of moistened cotton. At the time of urination, the color should be observed (important in the assessment of dehydration), as previously described (see assessment of the neonatal triad–dehydration), as well as the presence of hematuria, which is common in neonatal sepsis and feline neonatal isoerythrolysis. The neonatal urine output is 0.1 mL per 100 g of weight per hour, and oliguria and anuria are common in severe dehydration, septic shock, and malformations [2,10,54].
- Assessment of the anus and feces: to observe the feces, stimulation of defecation must be performed with gentle massage of the anus with the help of moistened cotton. The first feces expelled by the newborn are called meconium and are brownish. After starting breastfeeding, the newborn’s feces become yellowish. Color changes, such as a greenish or whitish color or the presence of live or digested blood, should be observed. Furthermore, the meconium and feces of a healthy newborn must be consistent and must not be pasty or diarrheal (Figure 25 and Figure 26). These changes are frequently associated with gastrointestinal disorders, such as bacterial, parasitic, or viral infections and nutritional or management errors. Diarrhea is the main clinical sign of parasitic and bacterial infection, occurring in approximately 93% of cases of neonatal sepsis in puppies. Meconium diarrhea is associated with neonatal systemic infection arising from intrauterine/maternal infection [44]. Constant diarrhea can also cause proctitis, resulting in perianal hyperemia. Another common change in the neonatal period is dry feces, culminating in constipation. This condition occurs mainly due to dehydration and is common in management errors of orphaned newborns when water is not administered between breastfeeding. For monitoring, it is important to know that newborns should defecate at least twice a day [2].
- Malformations: Anal atresia, rectovaginal fistula, vaginal atresia, hypospadias, among others, can commonly be observed. Atresias require immediate surgical correction.

##### Limbs and Tail

To evaluate the limbs and tail, the presence of trauma, injuries, and malformations must be observed.

**Figure 27:**
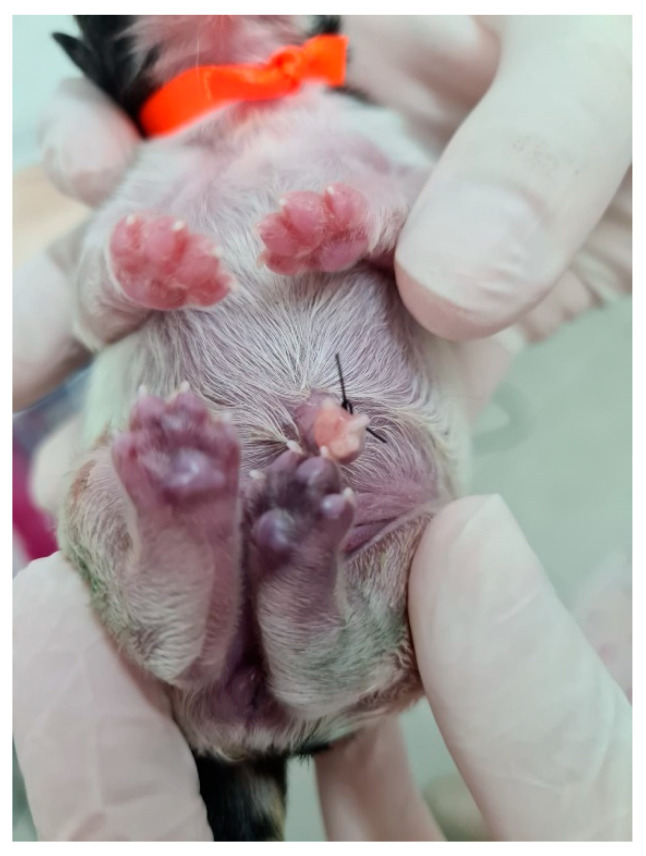
Cyanosis in the pelvic limbs in a neonatal puppy with sepsis.

**Figure 28:**
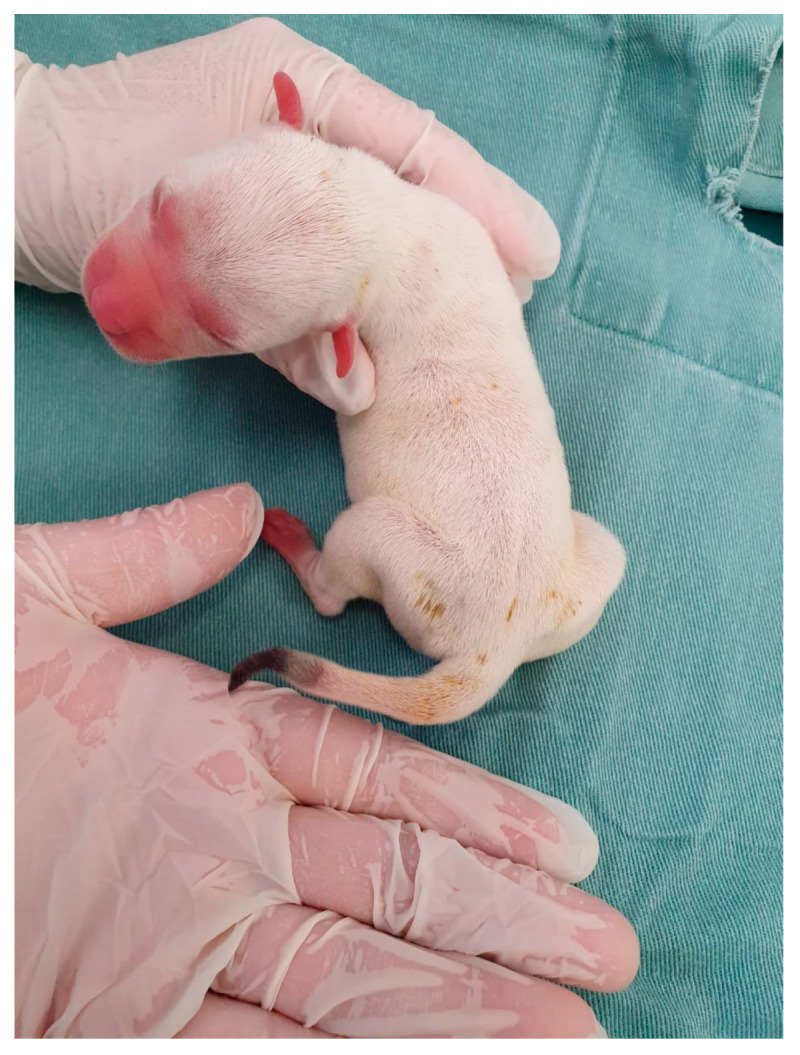
Tail tip necrosis in a neonatal puppy with sepsis.

**Figure 29:**
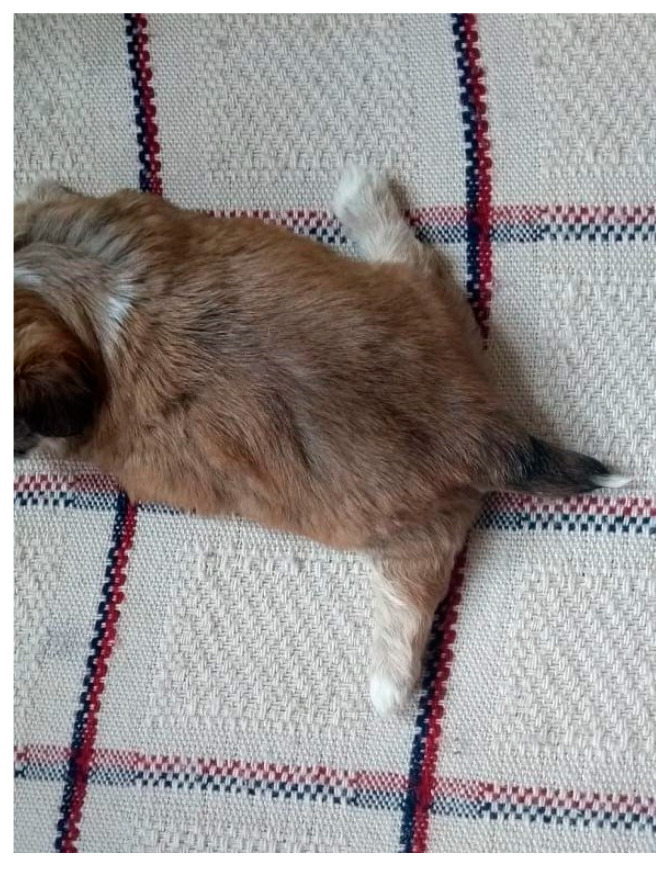
Swimming puppy syndrome in pelvic limbs.

- Traumas and lesions: traumas due to failure in maternal instinct, such as lesions and amputation of the limbs and tail, can occur in neonatal routine. Another observed change is cyanosis of the limb, digit, or tail extremities (Figure 27), which frequently occurs in neonatal sepsis and feline neonatal isoerythrolysis, which can progress to necrosis of the extremities (Figure 28) if not diagnosed and treated early. Cyanosis occurs as a result of hypoxia due to reduced blood supply to the extremities (due to systemic vasodilation and hypotension) associated with vasculitis caused by the infectious agent [44,53]. Another change observed in the limbs is an increase in volume, as in cases of abscesses and osteomyelitis.
- Malformations: in the limbs and tail, we can diagnose congenital defects, such as amelia (absence of limb), pygomyelia (increased number of limbs), meromyelia (absence of the end of limbs), swimmer cub syndrome (abduction of limbs) (Figure 29), equinovarus (twisted limb), polydactyly (larger number of digits), sidactyly (absence of digits), a twisted tail, and absence of the tail, among others.

##### Skin

Skin evaluation is based on the observation of lesions that may indicate dermatopathies, the presence of ectoparasites, and congenital changes.

**Figure 30:**
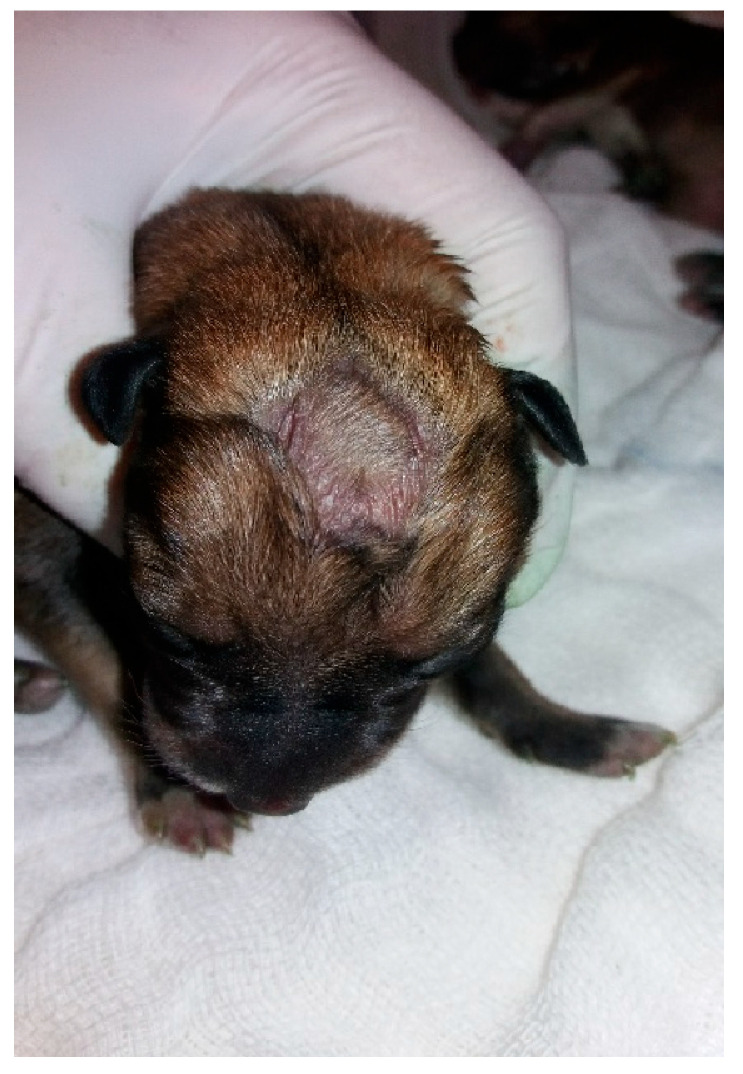
Aplasia cutis in a neonatal puppy.

**Figure 31:**
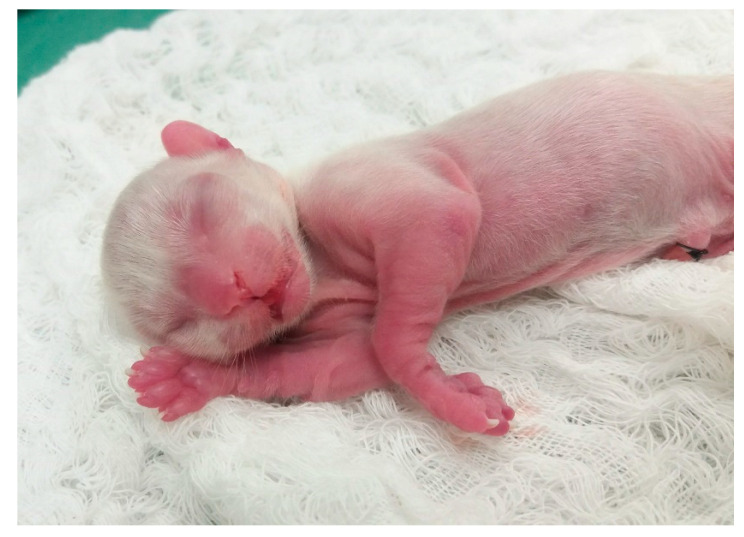
Absence of fur on the extremities of a premature neonatal kitten.

- Skin lesions: signs of bacterial, parasitic, fungal, and other skin diseases can be observed, such as alopecia, crusts, papules, pustules, abscesses, vesicles, erythema, erosion, scaling, dry, moist, or oily skin.
- Ectoparasites: assess the presence of fleas, ticks, lice, and larvae (myiasis) and carry out complementary tests to diagnose scabies.
- Congenital or genetic changes included aplasia cutis (absence of the formation of regions of skin/fur) (Figure 30), which is frequently observed in the head region; skin asthenia (hyperextensibility and skin fragility); juvenile cellulitis; alopecia due to color dilution; and ichthyosis, among others. In premature newborns, we can observe the absence of fur on the ends of the limbs, face, and tail (Figure 31) due to the shorter time of intrauterine development.

#### 3.3.7. Neonatal Weighing

Neonatal weighing is one of the main newborn management strategies, essential for identifying newborns at risk. Steady weight gain is an indication of neonatal health and well-being. Healthy newborns who are breastfeeding adequately should gain weight every day, expecting a minimum weight gain of 5 to 10% daily [2,11,55], which doubles their weight in seven to 10 days. In puppies, the daily growth rate can vary according to the size of the breed: 5 to 7% for small breeds, 8 to 10% for medium breeds, and 5 to 9% for large/giant breeds [unpublished data].

Weight loss, or insufficient gain (<5%), is related to a breastfeeding problem (maternal agalactia/hypogalactia, failure in maternal care, absent or weak neonatal sucking, large litter, among others), management errors, or some neonatal disease, since any affection can lead to neonatal clinical depression, apathy, reduced sucking reflex and consequent reduction in milk intake, which will culminate in weight loss. Often, failure to gain weight occurs before the development of clinical signs of disease; thus, a newborn who is at risk can be identified early, and diagnosis and intervention can be carried out, which is essential for a greater chance of survival [11,55].

Although a weight loss of up to 10% within 24 h after birth is commonly described in the literature as physiological and tolerable in puppies, studies attempt to dispel this myth [4,41,43]. In fact, in healthy newborns, this weight loss is not observed in the neonatal routine. When this occurs, it is related to some change or disease, as described previously. According to Mila et al. (2017), negative growth rates during the first two days of life are linked to a greater risk of mortality in the first 21 days of life. The initial growth rate cutoff value that defines puppies at risk is −4%. Approximately 40% of puppies with retarded growth die during the neonatal period, whereas only 5% of puppies with adequate growth die. The absence of weight gain may suggest insufficient colostrum intake, which is essential for newborn puppies, both for maintaining caloric requirements and for the transfer of passive immunity [4,43]. Furthermore, a study demonstrated that the colostrum microbiota can vary according to the type of parturition, impacting the growth rate of the puppies. The colostrum of bitches and the meconium of puppies from vaginal birth and elective cesarean section demonstrated a significantly higher abundance of bacteria compared to emergency cesarean section. Consequently, puppies with greater microbiota presented greater weight gain in the first days of life [56].

**Figure 32:**
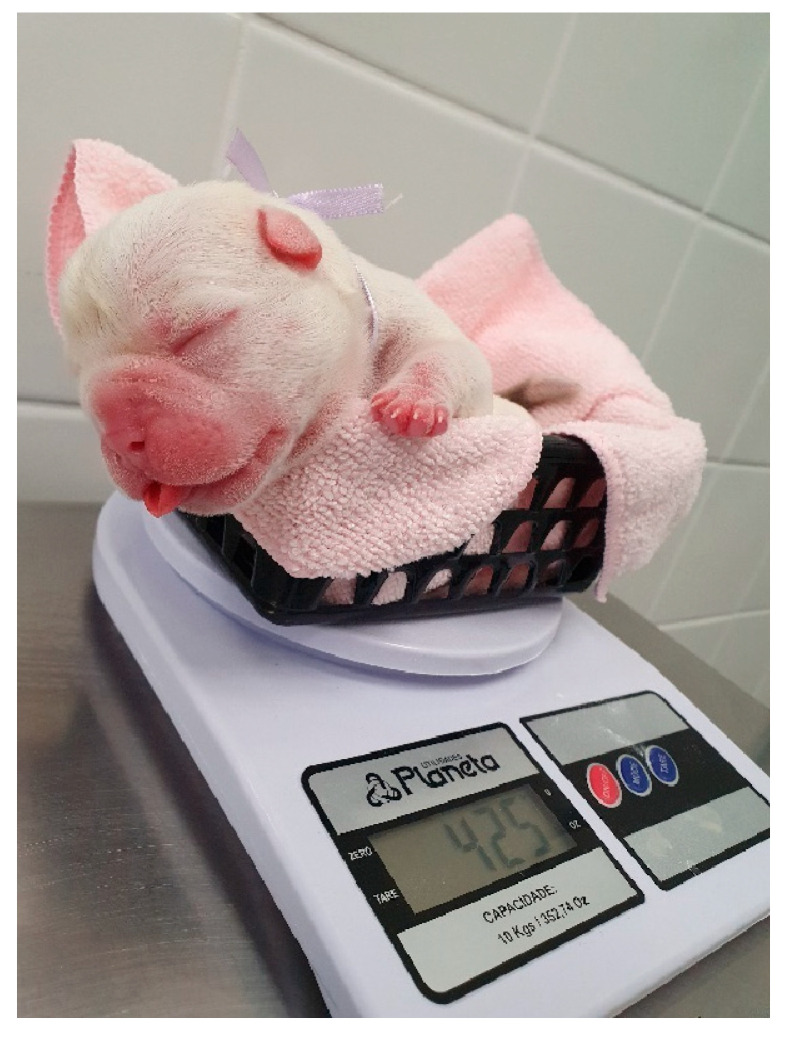
Neonatal weighing on a small digital scale in grams.

The daily weight gain of newborns must be assessed using a small digital scale in grams (Figure 32). The weight must be recorded on evaluation sheets and can be easily carried out by tutors or breeders.

**Figure 33:**
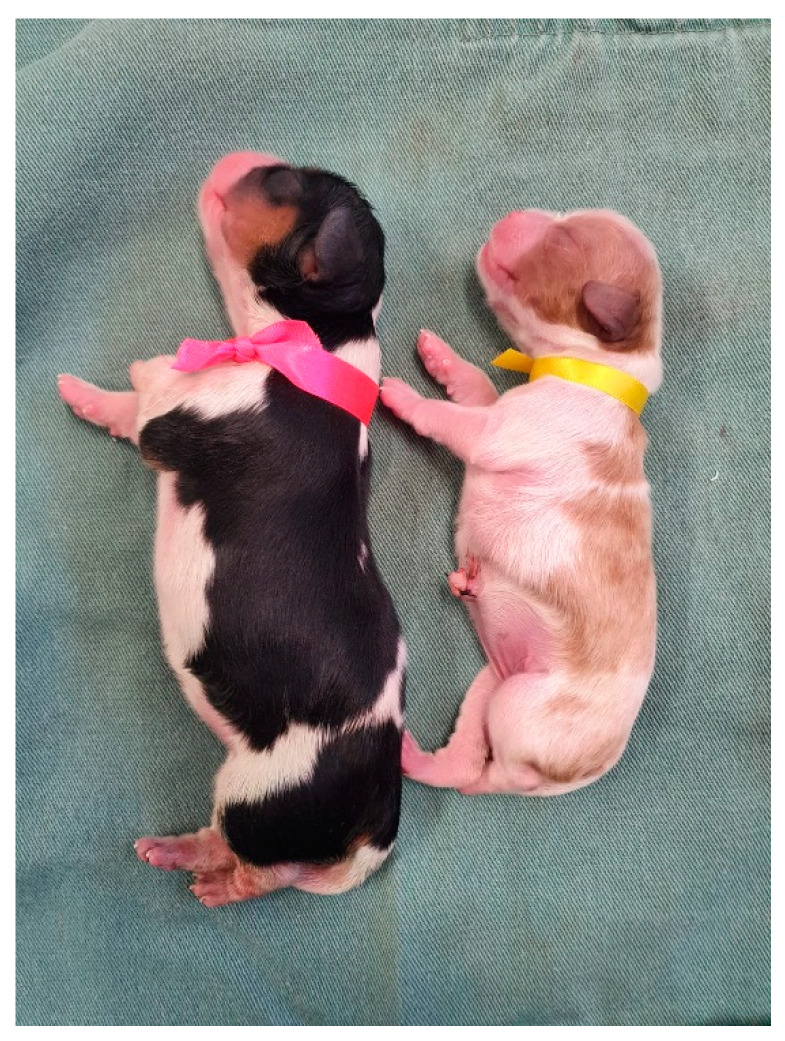
Low-birth-weight puppy (**right**) compared to his littermate (**left**).

A risk group for mortality is low-birth-weight newborns (Figure 33). These newborns suffered intrauterine growth restriction due to deprivation of adequate oxygenation and nutrition resulting from placental changes. They are characterized by being born at least 25% lighter than the average weight of their littermates or their breed. Consequently, they are physiologically more immature and susceptible to lower viability at birth, lower vitality scores, weak sucking reflex, immune deficits, lower hepatic glycogen stores, greater deficiency of the thermoregulatory system, and a higher risk of developing neonatal triad, fading syndrome, and infections [5,8,43].

For these reasons, weight is of great relevance in predicting neonatal mortality; more than 80% of puppies who die in the first 48 h of life (early mortality) are low-birth-weight newborns [43], and approximately 60% of low-birth-weight kittens do not survive weaning [2], especially if they do not have adequate assistance. Therefore, the identification of low-birth-weight newborns and the appropriate management of these patients are crucial for their survival. Birth weight can be analyzed according to each size due to the wide variation in body weight (Table 8). However, studies have shown that there are size variations between breeds [5,8] (Table 9).

Furthermore, weight limits were established for these at-risk newborns, namely, low birth weight (LBW) and very low birth weight (VLBW), where their classification depends on the species and race (Table 10 and Table 11). These two thresholds distinguish newborns at slightly higher risk and those at very high risk of death compared with normal birth weight (NBW) pups. Puppies with NBW have neonatal mortality of 4% (during the first 21 days of life); for LBW puppies, it is 9%; and for VLBW puppies, it is up to 55% [57]. Kittens with NBW present 4.5% mortality during the first two months of life, whereas those with LBW are 16.5%, and those with MLB are 50.9% [8].

These practical guidelines for measuring weight provide an easy tool for detecting and nursing puppies at increased risk of mortality. They may be relevant for breeders and veterinarians to objectively identify precocious puppies that require special attention, reducing the mortality rate [8].

Low birth weight in canines and felines still has a poorly understood and insufficiently investigated etiology. Due to the current scarcity of studies on risk factors and how to prevent them, measures to date have been limited to providing breastfeeding care and monitoring to improve the survival rates of these puppies and kittens [8]. More research is essential to study the etiology and medium- and long-term consequences of low birth weight.

#### 3.3.8. Facts and Periods of Neonatal Development

For an appropriate clinical approach, veterinarians must be aware of the maturation time or development of the newborn’s systems or organs to identify the possible age of the newborn when this information is not available and to guide or resolve the doubts of tutors and breeders on monitoring the development of the litter. Table 12 shows the main facts of puppy and kitten neonatal development and the period in which they occur.

#### 3.3.9. The Role of the Owner in Identifying Newborns at Risk

It is important to instruct the owner to constantly evaluate their litters and when to consult a veterinarian if any abnormalities are observed. They must be instructed to monitor weight gain, stimulate urination and defecation to observe the color and consistency of excreta, observe the behavior of the litter and the mother, whether the puppies are suckling, milk the breasts to observe production and the appearance of the milk, assess the neonatal temperature with a digital or infrared thermometer, observe the respiratory pattern, and observe the color of the oral region, abdomen, umbilical region, and limb extremities.

Litter daily monitoring can ensure early identification of clinical signs of changes or affections. Table 13 shows the main risk signs that owners can observe, and based on these findings, the litter and mother can be referred for a veterinary clinical appointment.

### 3.4. Complementary Exams

After the neonatal physical examination, additional tests, such as complete blood count, biochemistry, urine, coproparasitology, radiography, ultrasound, echocardiogram, electrocardiogram, blood gas analysis, and lactate, may be requested to aid in the diagnosis. Complementary exam results must be interpreted according to the newborn dogs’ and cats’ physiological characteristics and reference parameters. However, studies on the laboratory parameters of newborn puppies and kittens are limited; some of them are old, and updating is always necessary.

#### 3.4.1. Laboratory Analyses

To perform exams, the newborn must be positioned under a heated surface to prevent hypothermia. Long periods of fasting are not possible in neonatal patients due to the risk of hypoglycemia.

Blood samples can be obtained from the jugular vein using 24- or 26-gauge needles coupled with 1 to 3 mL syringes. The blood volume of newborns is approximately 5 to 8 mL/100 g of body weight. The blood sample collected must be less than 1 mL/100 g of weight in 24 h. The minimum recommended laboratory parameters include blood glucose, hematocrit, total protein, and leukocyte count [2,55,58]. Physical restraint for collection can be carried out with the newborn in lateral or dorsal decubitus, with the front limbs close to the chest and abdomen and the neck and head extended. For collection, the authors prefer to insert the needle into the jugular vein in a cranio-caudal direction (Video S12).

Neonates have physiological particularities that reflect the interpretation of red blood cell count results. Considering the body’s compensatory response to physiological hypoxemia during the birth process, at birth, they have a higher hematocrit, polycythemia, macrocytosis, a higher concentration of reticulocytes, and polychromasia. However, the number of red blood cells begins to decrease from the third day of age due to the replacement of fetal red blood cells by neonatal red blood cells, culminating with a decrease in hematocrit, known as physiological anemia of the newborn [1,58]. It is important to note that the reference values for red blood cells and leukocytes differ among adult animals and change according to neonatal age, as well as according to species (Table 14 and Table 15).

In the biochemical profile, physiological particularities are also observed during the neonatal period (Table 16). The alanine aminotransferase (ALT) and total protein (mainly albumin) levels are reduced compared to adults due to liver immaturity. The levels of alkaline phosphatase (ALP) and gamma-glutamyl transpeptidase (GGTP) are 30 to 100 times greater than those in adults during the first 10 days of life. This elevation occurs after the ingestion of colostrum, which is rich in these enzymes, which allows them to be used as an indirect assessment of failure in the transfer of passive immunity. Urea, on the other hand, varies rapidly with food intake during the weeks of life but is still a more accurate indicator of kidney abnormalities than creatinine, which is reduced in newborns (compared to adults) due to its lower muscle mass [1,59]. Other tests commonly used to evaluate the clinical condition of the newborn at birth, such as blood gas analysis, oxygen saturation, troponin I, and lactate, are mainly performed in cases of perinatal asphyxia/hypoxia resulting from cases of dystocia, prolonged births, and cesarean sections (Table 17) [21,33].

Urine samples can be collected by stimulating the micturition reflex. The minimum urinalysis assessment includes the determination of specific density and urinary sediment. Normal urinary density is 1.006 to 1.017 until the eighth week of age. Physiological proteinuria and glycosuria may be present in the first few days of life due to renal immaturity and low glomerular filtration rate. Stool samples for coproparasitology can be obtained by stimulating the defecation reflex [1,2,33].

**Table 14:** Mean, minimum, and maximum blood cell count parameters of neonatal puppies according to age (adapted from [58]).

	Age (Weeks)				
Parameter	At Birth	1	2	3	4
Red blood cells (×10^6^/µL)	5.1 (4.7–5.6)	4.6 (3.6–5.9)	3.9 (3.4–4.4)	3.8 (3.5–4.3)	4.1 (3.6–4.9)
Hemoglobin (g/dL)	15.2 (14.0–17.0)	12.9 (10.4–17.5)	10.0 (9.0–11.0)	9.7 (8.6–11.6)	9.5 (8.5–10.3)
Hematocrit (%)	47.5 (45.0–52.5)	40.5 (33.0–52.0)	31.8 (29.0–34.0)	31.7 (27.0–37.0)	29.9 (27.0–33.5)
MCV (µ^3^)	93.0	89.0	81.5	83.0	73.0
MCHC (%)	32.0	32.0	31.5	31.0	32.0
Reticulocytes (%)	6.5 (4.5–9.2)	6.9 (3.8 a 15.2)	6.7 (4.0–8.4)	6.9 (5.0–9.0)	5.8 (4.6–6.6)
Leukocytes (×10^3^/µL)	12.0 (6.8–18.4)	14.1 (9.0–23.0)	11.7 (8.1–15.1)	11.2 (6.7–15.1)	12.9 (8.5–16.4)
Neutrophils	8.6 (4.4–15.8)	7.4 (3.8–15.2)	5.2 (3.2–10.4)	5.1 (1.4–9.4)	7.2 (3.7–12.8)
Lymphocytes	1.9 (0.5–4.2)	4.3 (1.3–9.4)	3.8 (1.5–7.4)	5.0 (2.1–10.1)	4.5 (1.0–8.4)
Monocytes	0.9 (0.2–2.2)	1.1 (0.3–2.5)	0.7 (0.2–1.4)	0.7 (0.1–1.4)	0.8 (0.3–1.5)
Eosinophils	0.4 (0.0–1.3)	0.8 (0.2–2.8)	0.6 (0.08–1.8)	0.3 (0.07–0.9)	0.25 (0.0–0.7)
Basophils	0.0	0.01 (0.0–0.2)	0.0	0.0	0.01 (0.0–0.15)
Platelets (×10^3^/µL)	178–465	282–560	210–352	203–370	130–360

MCV = mean corpuscular volume; MCHC = mean corpuscular hemoglobin concentration.

**Table 15:** Mean blood cell count parameters of neonatal kittens according to age (adapted from [60]).

	Age (Weeks)		
Parameter	0 to 2	2 to 4	4 to 6
Red blood cells (×10^6^/µL)	5.29 ± 0.24	4.67 ± 0.10	5.89 ± 0.23
Hemoglobin (g/dL)	12.1 ± 0.6	8.7 ± 0.2	8.6 ± 0.3
Hematocrit (%)	35.3 ± 1.7	26.5 ± 0.8	27.1 ± 0.8
MCV (µ^3^)	67.4 ± 1.9	53.9 ± 1.2	45.6 ± 1.3
MCHC (%)	34.5 ± 0.8	33.0 ± 0.5	31.9 ± 0.6
Reticulocytes (%)	9.67 ± 0.57	15.31 ± 1.21	17.45± 1.37
Leukocytes (×10^3^/µL)	5.96 ± 0.68	6.92 ± 0.77	9.57 ± 1.65
Neutrophils	3.73 ± 0.52	6.56 ± 0.59	6.41 ± 0.77
Lymphocytes	0.01 ± 0.01	0.02 ± 0.02	0.00
Monocytes	0.96 ± 0.43	1.40 ± 0.16	1.47 ± 0.25
Eosinophils	0.02 ± 0.01	0.00	0.00

MCV= mean corpuscular volume; MCHC= mean corpuscular hemoglobin concentration.

**Table 16:** Minimum and maximum biochemical parameters of neonatal puppies and kittens according to age (adapted from [59,61]).

	Puppies			Kittens		
Parameter	1–3 Days	2 Weeks	4 Weeks	1 Day	2 Weeks	4 Weeks
Total bilirubin (mg/L)	0.2–1.0	0.1–0.5	0.0–0.1	0.1–1.6	0.1–1.0	0.1–0.2
ALT (IU/L)	17–337	10–21	20–22	7–42	11–24	14–26
AST (IU/L)	45–194	10–40	14–23	75–263	8–48	12–24
ALP (IU/L)	618–8.760	176–541	135–201	1348–3715	68–269	90 -135
GGTP (IU/L)	163–3.558	4–77	2–7	0–9	0–3	0–3
Urea	23–37	15–23	10 -21	34–94	22–54	17–30
Creatinine	0.4–0.6	0.3–0.5	0.3–0.5	0.6–1.2	0.2–0.6	0.3–0.5
Total protein (g/dL)	3.4–5.2	3.6–4.4	3.9–4.2	3.9–5.8	4.0–5.2	4.6–5.2
Albumin (g/dL)	1.5–2.8	1.7–2.0	1.0–2.0	1.9–2.7	2.0–4.0	2.2–2.4
Cholesterol (mg/dL)	112–204	223–344	266–352	48–212	164–443	222–434
Calcium (mg/dL)	10.4–13.6	11.2–13.2	10.4–13.2	9.6–12.2	10.0–13.7	10.0–12.2
Phosphorus (mg/dL)	5.26–10.83	8.35–11.14	8.66–11.45	4.90–8.90	6.70–11.00	6.70–9.00

ALP = alkaline phosphatase; ALT = alanine aminotransferase; AST = aspartate aminotransferase; GGTP = gamma-glutamyl transpeptidase.

**Table 17:** Mean, minimum, and maximum values of the parameters troponin I, venous blood gas analysis, lactate, and oxygen saturation of neonatal puppies at birth and 60 min later (adapted from [33]).

Parameter	Time Point	Type of Parturition/Clinical Condition		
		Eutocia	Cesarean Section	Perinatal Asphyxia
Troponina I (ng/mL)	At birth 60 min after	0.02 (<0.01–0.04) 0.01 (<0.01–0.03)	0.05 (0.03–0.07) 0.05 (0.02–0.08)	0.15 (0.11–0.25) 0.11 (0.07–0.20)
pH	At birth 60 min after	7.1 (7.0–7.3) 7.2 (7.1–7.3)	7.2 (7.1–7.3) 7.3 (7.2–7.3)	7.1 (6.9–7.2) 7.3 (7.2–7.3)
pCO_2_ (mmHg)	At birth 60 min after	43.2 (29.2–58.2) 46.8 (33.2–60.8)	49.3 (39.0–59.9) 48.9 (45.7–52.5)	64.4 (55.9–71.7) 52.8 (47.8–60.2)
pO_2_ (mmHg)	At birth 60 min after	21.7 (13.0–36.0) 21.4 (17.0–35.0)	13.7 (8.0–19.0) 14.7 (12.0–17.0)	7.0 (5.0–9.0) 11.0 (10.0–12.0)
HCO_3_ (mmol/L)	At birth 60 min after	16.5 (8.5–22.0) 21.7 (16.5–26.9)	23.2 (19.8–27.1) 25.1 (23.0–26.5)	25.7 (19.9–32.5) 27.5 (23.8–31.4)
TCO_2_ (mM)	At birth 60 min after	18.0 (10.0–24.0) 23.4 (18.0–29.0)	25.1 (21.0–29.0) 26.9 (25.0–28.0)	28.2 (22.0–35.0) 29.4 (26.0–33.0)
Beecf (mmol/L)	At birth 60 min after	−12.5 (−23.0 to −6.0) −5.8 (−10.0 to −1.0)	−5.1 (−8.0 to −1.0) −1.7 (−4.0 to 0.0)	−4.2 (−13.0 to 4.0) 0.4 (−4.0 to 4.0)
Lactate (mg/dL)	At birth 60 min after	2.3 (1.7–2.7) 1.8 (1.5–2.2)	3.9 (2.3–5.5) 2.3 (1.8–3.2)	4.8 (2.2–9.0) 2.1 (1.4–2.9)
SO_2_ (%)	At birth 60 min after	31.4 (11.0–57.0) 33.2 (20.0–48.0)	19.6 (6.0–36.0) 19.9 (15.0–30.0)	8.6 (4.0–12.0) 14.0 (10.0–18.0)
Peripheral SO_2_ (%)	At birth 60 min after	91.6 (65.0–99.0) 99.0	97.9 (94.0–96.0) 99.0	57.6 (48.0–74.0) 98.0

#### 3.4.2. Imaging Exams

##### Radiographic Examination

**Figure 34:**
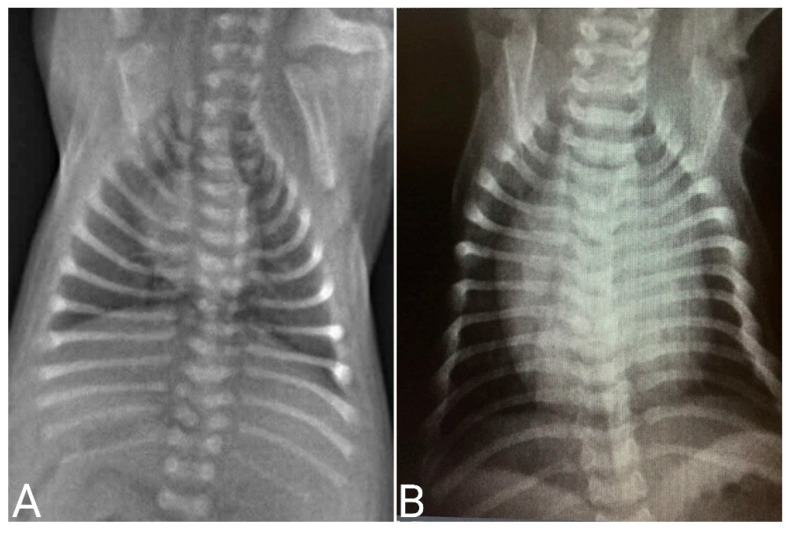
Thorax radiographs in neonatal puppies. (**A**) Healthy neonate. (**B**) Neonate presenting cardiomegaly on radiographic examination due to congenital heart disease.

Radiographic interpretation also has particularities in neonatal patients. Little bone mineralization and the small thickness of soft tissues interfere with the quality of ray penetration. A decrease in kilovoltage (kV) to half that used in adult animals has been described. The absence of costochondral mineralization, open growth plates in long bones, loss of abdominal details (due to lack of body fat), and a more opaque lung interstitium due to increased water content in the parenchyma, and often, the heart may appear relatively large in proportion to the thorax [10,25]. Therefore, when the veterinarian is not familiar with the physiological aspects of neonatal radiographs, we suggest that radiographs be taken of a healthy littermate, together with the affected neonate, as a way of comparing the physiological pattern and possible changes (Figure 34). Radiographic examination is commonly requested to identify congenital alterations, cardiopulmonary alterations, foreign bodies, traumas, and orthopedic alterations, among others.

##### Ultrasound Examination

**Figure 35:**
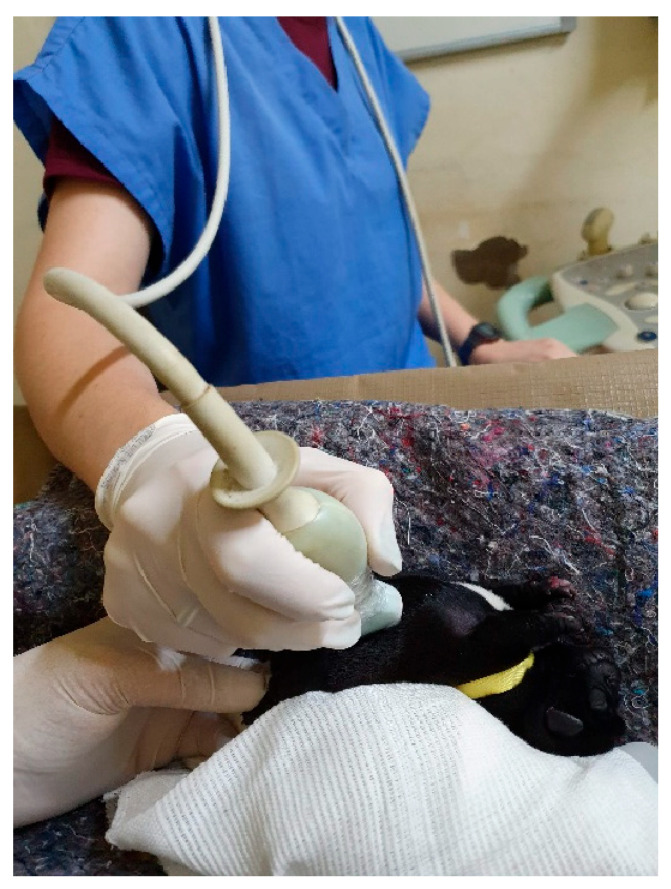
Abdominal ultrasound in a neonatal puppy.

Ultrasonography of the abdominal cavity of neonatal patients is performed using 6- to 8-MHz transducers (Figure 35). The most frequently found artifacts are acoustic shadows due to the greater amount of food and gases in the digestive system, which can increase the difficulty of adequately visualizing organs such as the liver and other viscera. In newborns, a lack of a full urinary bladder and a greater occurrence of abdominal fluid are common. Oppositely to radiographic examination, the smaller amount of abdominal fat provides better image quality [2,24]. Ultrasound examination is often requested to identify congenital anomalies, foreign bodies, constipations, parasitic infections, and signs of infectious diseases.

##### Echocardiographic Examination

**Figure 36:**
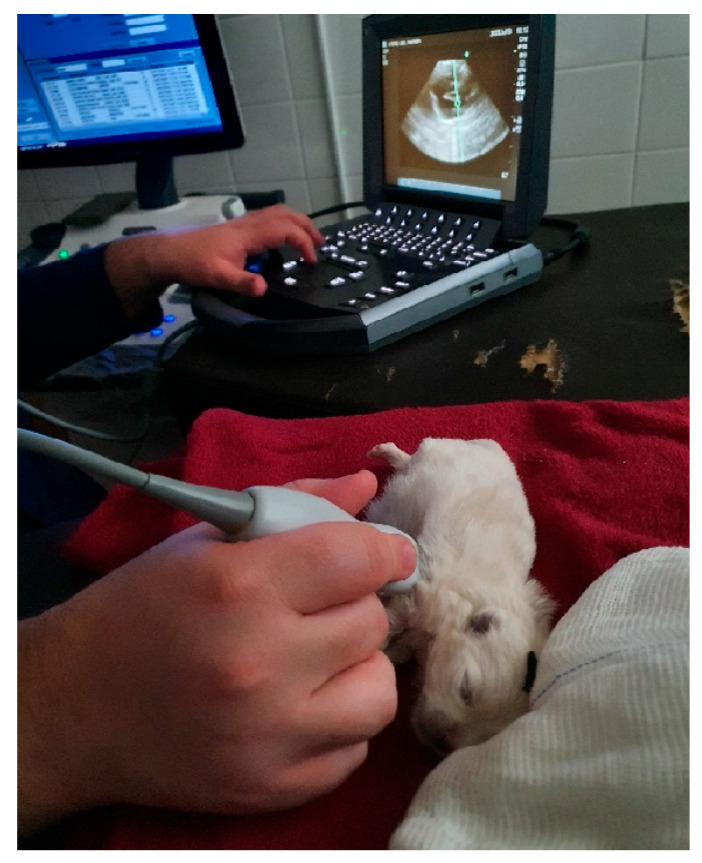
Echocardiogram in a neonatal puppy.

For the echocardiographic examination (Figure 36), the newborn must be positioned in the lateral decubitus on a heated surface, and the ultrasound gel must also be previously heated to provide a more comfortable environment. The transducer is positioned on the left chest wall, and depending on its dimensions, images are obtained through several intercostal spaces simultaneously. Using pulsed Doppler, peak blood flow speeds are measured through the mitral, tricuspid, aortic, and pulmonary valves. Color flow Doppler imaging is of great value for observing and identifying flows and helps to position the cursor to measure peak flow velocity. The flow direction is identified based on their coding in red or blue, which is crucial for understanding the hemodynamics of the newborn’s small heart. With echocardiography, it is possible to diagnose several congenital heart diseases that affect canine and feline newborns.

##### Electrocardiographic Examination

**Figure 37:**
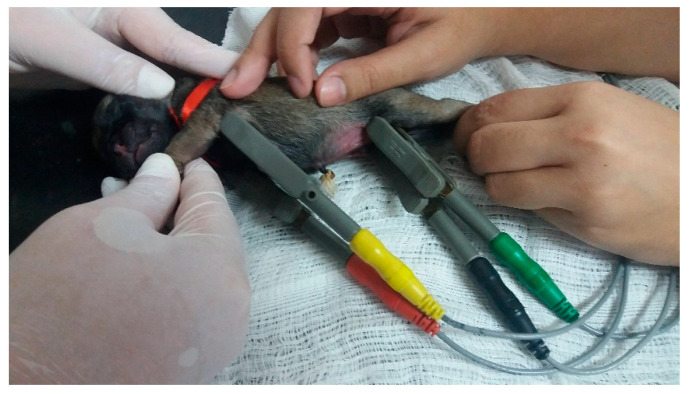
Electrocardiogram in a neonatal puppy.

With an electrocardiogram (Figure 37), it is possible to diagnose cardiac arrhythmias and/or electrical conduction disorders, being a practical method, and its use is viable in the neonatal period. To perform this exam, the newborn must be positioned in the right lateral decubitus position on a soft, heated surface, and the electrodes must be placed above the elbow and knee joints. It is advisable to wait until the newborn settles in and thus carry out the electrocardiographic examination calmly without much interference at baseline. During the first 30 days of life, variations in the amplitudes of the electrocardiographic waves Q, R, and S, changes in the orientation of the electrical axis, and in the R/S ratio are observed in puppies and kittens. Such changes demonstrate that during the first month of a newborn’s life, there is a change in the predominance of the right ventricle over the left ventricle [20,26]. Regarding the influence of the type of delivery, a study demonstrated that during the first 35 days of life, newborn puppies from emergency cesarean sections had lower heart rate variability than newborns from eutocic birth, with consequent delays in the autonomic influence on the heart. Concluding that the type of birth must be considered when evaluating the activity of the autonomic nervous system in newborns [20].

In small animal neonatology, reference standards for some complementary exams may not be available yet, providing a vast field for new research. It is important to emphasize that despite the various neonatal particularities, complementary exams are feasible for these patients and should be carried out when necessary.

## 4. Conclusions

Dogs and cats are altricial species, immature and dependent on the mother to survive the neonatal period. Several organic systems are under development, making the clinical assessment of neonatal patients different from adult animals in clinical, laboratory, and imaging. Veterinarians must be familiar with the physiological, anatomical, hematological, and biochemical neonates’ characteristics, recognizing sick patients and providing necessary assistance. Despite some limitations, the clinical examination of newborns can be effectively performed and should not be neglected. In general, to recognize a newborn at risk, it is necessary to carefully and constantly monitor the litter and the mother. Knowing the key points of healthy newborns will help in recognizing the sick ones: Healthy newborns have strong reflexes and muscle tone; continually gain weight; have consistent stools and defecate at least twice a day; have light yellow or transparent urine; after breastfeeding, they remain resting and do not vocalize constantly; newborns breathe with their mouth closed and have a regular breathing pattern; have an umbilical region free of erythema or purulent secretion; and have a pinkish body color.

## 5. Future Directions

Clinical, laboratory, and imaging parameters of neonatal puppies and kittens continue to be investigated. Data on the evolution of reference parameters over weeks of life are scarce, especially in kittens. Furthermore, to interpret what is normal in newborns, some physiological aspects still need to be characterized. For example, in the puppy, the neonatal kidney is morphologically and functionally immature; nephrogenesis continues for at least two weeks after birth [46]. However, there is a lack of information about nephrogenesis in cats. Consequently, there are only assumptions about the renal structure of feline newborns and some parameters, such as urine-specific gravity [1].

To fill in the gaps regarding physiology and normality parameters, more studies are needed. The main research needs are related to the maturity of systems, such as cardiovascular, respiratory, urinary, and immune systems, and the evolution of physical examination reference data during the neonatal period since most of them are focused on the moment after birth, such as heart rate, respiratory rate, blood pressure, blood glucose, temperature, and hydration. Updating hematological and biochemical parameters during the neonatal period is also necessary; some studies are old, and others are limited, with a lack of lymphocyte, eosinophil, monocyte, platelet, GGT, sodium, potassium, calcium, or phosphorus counts [58,60,62,63] or evaluating parameters only from 16 days of age [64]. Additionally, possible variations in the values of these exams between small, medium, large, and giant dogs should be investigated.

Studies on the measurements and aspects of imaging exams in newborns and their variations by race are essential. There are frequent doubts among clinicians about what would be normal or abnormal when evaluating neonatal radiographs, ultrasounds, or echocardiograms of puppies and kittens.

Knowing the reference parameters and how to carry out clinical assessment on the basis of specific studies is crucial for determining the appropriate time to initiate prophylactic and therapeutic approaches, impacting the reduction in neonatal mortality rates.

## Figures and Tables

**Table 1:** Key points about the clinical history and anamnesis of the neonatal patient.

Clinical History and Anamnesis		
**General identification**	Date of clinical appointment, name, species, breed, sex, age;	
	Name and address of the owner.	
**Main reason for a veterinary appointment**	Why did the owner bring the animal for veterinary care?	
**History/anamnesis**	**Points to be investigated**	**Importance of newborn care**
About the health and reproductive history of the parents	Vaccination; deworming; previous diseases; genetic tests performed; PCRs and serologies; Herpesvirus vaccines used/or not; use of drugs; primiparity; previous parturition; number of newborns from previous litters; type of parturition; abortion, dystocia, agalactia, mastitis, malformations, maternal behavior and mortality in previous litters; inbreeding; use of contraceptives.	Suspected infectious diseases and congenital defects in the litter; disturbances during parturition; investigation of the neonatal triad; prevention of early neonatal mortality.
About pregnancy	Nutrition; drugs or supplements administered to the mother; deworming during pregnancy; exposure to other animals; carrying out prenatal examinations; diseases during pregnancy; abortion.	Suspected teratogenic birth defects; investigation of early litter infections and mortality.
About parturition	Type of parturition; duration of parturition; whether the birth was full-term; dystocia; stillbirth or neonatal mortality; maternal care during parturition; immediate assistance during and after birth; umbilical cord rupture and disinfection; production, release, and intake of colostrum.	Investigation of early mortality; evaluation of cases of perinatal asphyxia; investigation of failure in maternal instinct/rejection; investigation of failure to transfer passive immunity and infections in the litter.
About the environment	Place of parturition; maternity; bed; hygiene, temperature and humidity control; contactants animals.	Investigation of maternal stress, rejection, litter hypothermia, and sources of infections.
About the newborn and the litter	Number of neonates; age; if the newborn is breastfeeding; body condition and birth weight; monitoring daily weight gain; isolated or littermate neonatal death; condition of the neonate before becoming ill; observed signs; orphanhood; colostrum intake; traumas; maternal stimulation for urination and defecation; aspect of feces and urine; vocalization; apathy; vomiting, diarrhea; change in breathing pattern; weight loss; previous treatments.	Assessment of clinical signs of neonatal disorders; investigation of the neonatal triad, failure to transfer passive immunity, underdevelopment, management errors, and infectious diseases.

**Table 2:** Basic materials used in neonatal clinical assessment.

Neonatal stethoscope or vascular Doppler
Digital rectal or infrared thermometer
Glucometer and lactimeter Pulse oximeter
Small digital balance, with scale in grams
Examination table with heated surface (thermal mattress, gloves with warm water, lamps, environmental heater, or other heating sources) Needles (24 and 26 gauge), 24 gauge catheters, syringes, and microtubes for blood collection (if there is a need to collect material for examination)
Disposable gloves
Neonatal clinical assessment form

**Table 3:** Vital parameters of puppies and kittens during the neonatal period.

Vital Parameters	Puppies	Kittens
Heart rate	200 to 260 bpm	200 to 280 bpm
Respiratory rate	15 to 40 mpm	40 to 160 mpm
Temperature		
1st week	35.0 to 37.2 °C	
2st 3st weeks	36.0 to 37.8 °C	
4st week	37.2 to 38.3 °C	
Blood pressure	50 to 70 mmHg	

Bpm: beats per minute; mpm: movements per minute.

**Table 4:** Modified Apgar score for neonatal puppies (adapted from [14,21]).

Parameters	0	1	2
**Heart rate**	<180 bpm	180–200 bpm	>220 bpm
**Respiratory rate**	Absent or <6 mpm	Weak and irregular <15 mpm (6–15)	Regular >15 mpm
**Muscle tone**	Flaccid	Some limb flexion	Flexion
**Reflex irritability**	Absent	Some movement	Hyperactivity or evident crying
**Mucous membrane color**	Cyanotic	Pale	Pink

Bpm = beats per minute; mpm = movements per minute.

**Table 5:** Modified Apgar score for neonatal kittens (adapted from [16]).

Parameters	0	1	2
**Heart rate**	<100 bpm	<180 bpm	200–280 bpm
**Respiratory rate**	Absent or <10 mpm	Weak and irregular <40 mpm	Regular 40–160 mpm
**Muscle tone**	Flaccid	Some limb flexion	Flexion
**Reflex irritability**	Absent	Some movement	Hyperactivity or evident crying
**Mucous membrane color**	Cyanotic	Pale	Pink

Bpm = beats per minute; mpm = movements per minute.

**Table 6:** Modified Apgar score and classification of neonatal viability of puppies (according to size) and kittens.

	Weak Viability/Severe Distress	Moderate Viability/Moderate Distress	Normal Viability/No Distress	
**Small breed dogs**	0–3	4	5–10	[34]
**Medium breed dogs**	0–3	4–5	6–10	
**Large breed dogs**	0–3	4–5	6–10	
**Cats**	0–3	4–6	7–10	[16]

**Table 7:** Neonatal reflex scores for puppies and kittens (adapted from [21,33]).

Neonatal Reflex	0	1	2
**Sucking**	Absent	Weak	Strong
**Rooting response**	Absent	Slow seeking for the mammary glands	Immediate seeking for the mammary glands
**Vestibular righting**	Absent (continues in the decubitus position)	Slow body repositioning	Immediate body repositioning

**Table 8:** Mean weight at birth of dogs and cats (adapted from [2]).

Species/Size	Weight
Cats	80–120 g
Small breed dogs	100–250 g
Medium breed dogs	250–350 g
Large breed dogs	350–500 g
Giant breed dogs	500–700 g

**Table 9:** Mean birth weight by breed of dogs and cats (adapted from [5,8]).

Dog Breeds	Size	Mean Birth Weight (g)	Cat Breeds	Mean Birth Weight (g)
Bichon Frisé	Small	189 (±37.5)	Abyssinian/Somali	97.2 (±11.8)
Cavalier King Charles Spaniel	Small	225.4 (±39.7)	Balinese/Mandarin/ Oriental/Siamese	95.4 (±13.1)
Chihuahua	Small	119.6 (±25.6)	Bengal	88.2 (±15.7)
Coton de Tulear	Small	187.9 (±35.5)	Birman	95.8 (±14.7)
Dachshund	Small	184 (±36.5)	British	98.4 (±17.1)
French Bulldog	Small	237.6 (±42.6)	Chartreux	110.4 (±18.5)
Jack Russell Terrier	Small	202.1 (±36.2)	Egyptian Mau	92.3 (±21.2)
Lhasa Apso	Small	187.5 (±40)	Maine Coon	119.1 (±18.7)
Maltese	Small	164.7 (±35.6)	Norwegian Forest	109.9 (±17.7)
Pomeranian	Small	152.1 (±40)	Persian/Exotic	85.5 (±15)
Shih Tzu	Small	176.4 (±27.9)	Ragdoll	100.3 (±13.5)
West Highland White Terrier	Small	196.3 (±37.5)	Russian Blue/Nebelung	92.7 (±15.2)
Yorkshire Terrier	Small	142.3 (±30.9)	Scottish/Highland	89.5 (±12.7)
Australian Shepherd	Medium	363 (±82)	Siberian	99.3 (±16.7)
Beagle	Medium	309 (±50.4)	Sphynx	90.3 (±14.6)
Cocker Spaniel	Medium	266.1 (±64.1)		
English Bulldog	Medium	315.9 (±68.1)		
Alaskan Malamute	Large	562.5 (±93.3)		
Boxer	Large	464 (±71.7)		
German Shepherd	Large	506.2 (±93.8)		
Golden Retriever	Large	395.4 (±71.7)		
Labrador Retriever	Large	410.2 (±69.7)		
Rottweiler	Large	403.8 (±58.6)		
White Swiss Shepherd	Large	473.4 (±80.7)		
Bernese Mountain dog	Giant	490.1 (±77.6)		
Leonberger	Giant	516.7 (±104.1)		
Newfoundland	Giant	630.3 (±112.1)		

**Table 10:** Classification of newborn puppies according to birth weight [57].

Breed	Normal Birth Weight (g)	Low Birth Weight (g)	Very Low Birth Weight (g)
Australian Shepherd	>375	213–375	<213
Bichon Frise	>181	163–181	<163
Cocker Spaniel	>280	142–280	<142
German Shepherd	>480	338–480	<338
Golden Retriever	>417	177–417	<177
Labrador Retriever	>406	248–406	<248
Maltese	>163	115–163	<115
Rottweiler	>410	345–410	<345
Shih Tzu	>176	128–176	<128
West Highland White Terrier	>190	129–190	<129

**Table 11:** Classification of newborn kittens according to birth weight [8].

Breed	Normal Birth Weight (g)	Low Birth Weight (g)	Very Low Birth Weight (g)
Abyssinian/Somali	>94	60–94	<60
Balinese/Mandarin/Oriental/Siamese	>82	78–92	<78
Bengal	>84	60–84	<60
Birman	>74	60–74	<60
British	>87	61–87	<61
Chartreux	>100	60–100	<60
Egyptian Mau	>104	61–104	<61
Maine Coon	>81	75–81	<75
Norwegian Forest	>94	60–94	<60
Persian/Exotic	>82	60–82	<60
Ragdoll	>84	60–84	<60
Russian Blue/Nebelung	>86	60–86	<60
Scottish/Highland	>77	60–77	<60
Siberian	>90	63–90	<63
Sphynx	>76	60–76	<60

**Table 12:** Neonatal development according to age (adapted from [2]).

Developmental Characteristic	Age (Days)	
	Puppy	Kitten
Pain/irritability reflex	At birth	At birth
March reflex	At birth	At birth
Sucking reflex	At birth	At birth
Breast seeking reflex	At birth	At birth
Vestibular righting reflex	At birth	At birth
Magnus reflex	At birth	At birth
Landau reflex	At birth	At birth
Flexor tone	1 to 4	1 to 4
Umbilical cord fallout	2 to 3	2 to 3
Extensor tone	5 to 21	5 to 21
Ability to crawl	7 to 14	7 to 14
Opening of the eyelids	12 to 15	8 to 12
Ear canal opening	12 to 17	12 to 15
Walking, urinating, and defecating spontaneously	15 to 21	15 to 21
Normal vision and threat reflex	21 to 30	21 to 30
Eruption of deciduous canine teeth	21 to 38	21 to 30
Eruption of deciduous incisor teeth	30 to 45	21 to 30
Thermoregulation similar to that of adults	28 to 30	45
Normal hearing	30 to 45	30 to 45
Eruption of deciduous premolar teeth	38 to 45	38 to 45
Kidney function similar to that of adults	55 to 60	50 to 60
Liver function similar to that of adults	120 to 150	120 to 150

**Table 13:** What signs should owners observe to identify newborns at risk?

- Weight loss
- Diarrhea or pasty feces
- Presence of blood or parasites in the feces
- Dark-colored urine or with the presence of blood
- Apathetic newborn
- Weak suction
- Flaccid muscle tone
- Cyanotic or pale mucous membranes
- Absence of breastfeeding
- Breathing with an open mouth
- Breathing pattern changing
- Hypothermia
- Erythematous abdominal or umbilical region
- Abdominal hematomas
- Cyanosis of the extremities of the limbs, digits, or tail
- Malformations
- Knowledge of trauma or evident lesion
- Seizures
- Growth retardation compared to littermates

## Data Availability

Not applicable.

## Supplementary Materials

The following supporting information can be downloaded at: https://www.mdpi.com/article/10.3390/ani14233417/s1, Video S1. Assessment of heart rate in a neonatal puppy with vascular Doppler. Video S2. Assessment of the respiratory rate in a neonatal kitten. Video S3. Apgar score in neonatal puppy. Video S4. Apgar score in a neonatal kitten. Video S5. Sucking, breast seeking and vestibular righting reflexes in a neonatal puppy. Video S6. Sucking, breast seeking and vestibular righting reflexes in a neonatal kitten. Video S7. March reflex together with the newborn’s seeking reflex. Video S8. Muscle tone in a neonatal kitten. Video S9. Convulsion in a neonatal puppy, demonstrating generalized muscle rigidity, followed by the return of the neonate’s movements after the seizure ceased. Video S10. Convulsion in a neonatal puppy demonstrating pedaling movements. Video S11. Respiratory distress in a neonatal puppy due to perinatal asphyxia and severe hypoxia. Video S12. Blood collection through the jugular vein in a neonatal puppy.
